# Interaction of SHP-2 SH2 domains with PD-1 ITSM induces PD-1 dimerization and SHP-2 activation

**DOI:** 10.1038/s42003-020-0845-0

**Published:** 2020-03-17

**Authors:** Nikolaos Patsoukis, Jonathan S. Duke-Cohan, Apoorvi Chaudhri, Halil-Ibrahim Aksoylar, Qi Wang, Asia Council, Anders Berg, Gordon J. Freeman, Vassiliki A. Boussiotis

**Affiliations:** 1 Division of Hematology-Oncology Beth Israel Deaconess Medical Center, Boston, MA 02215 USA; 2 Department of Medicine Beth Israel Deaconess Medical Center, Harvard Medical School, Boston, MA 02215 USA; 3 Department of Medical Oncology, Dana-Farber Cancer Institute, Harvard Medical School, Boston, MA 02215 USA; 4 Department of Pathology Beth Israel Deaconess Cancer Center, Harvard Medical School, Boston, MA 02215 USA; 5 Present Address: MD Anderson Cancer Center UTHealth Graduate School of Biomedical Sciences, Houston, TX 77030 USA; 6 Present Address: Cedars-Sinai Medical Center, Los Angeles, CA 90048 USA

**Keywords:** Enzymes, Immunotherapy, Cell signalling

## Abstract

Programmed cell death-1 (PD-1) inhibits T cell responses. This function relies on interaction with SHP-2. PD-1 has one immunoreceptor tyrosine-based inhibitory motif (ITIM) at Y223 and one immunoreceptor tyrosine-based switch motif (ITSM) at Y248. Only ITSM-Y248 is indispensable for PD-1-mediated inhibitory function but how SHP-2 enzymatic activation is mechanistically regulated by one PD-1 phosphotyrosine remains a puzzle. We found that after PD-1 phosphorylation, SHP-2 can bridge phosphorylated ITSM-Y248 residues on two PD-1 molecules via its amino terminal (N)-SH2 and carboxyterminal (C)-SH2 domains forming a PD-1: PD-1 dimer in live cells. The biophysical ability of SHP-2 to interact with two ITSM-pY248 residues was documented by isothermal titration calorimetry. SHP-2 interaction with two ITSM-pY248 phosphopeptides induced robust enzymatic activation. Our results unravel a mechanism of PD-1: SHP-2 interaction that depends only on ITSM-Y248 and explain how a single docking site within the PD-1 cytoplasmic tail can activate SHP-2 and PD-1-mediated inhibitory function.

## Abstract (web summary)

Patsoukis et al identify a mechanism by which SHP-2 phosphatase bridges two molecules of the inhibitory checkpoint receptor PD-1, and show this can also induce SHP-2 activation. These data provide insights into the mechanism of SHP-2 activation by PD-1 that may be relevant for its role in T-cell inhibition.

## Introduction

PD-1 is an inhibitory checkpoint receptor of the B7-CD28 family expressed in activated T cells. It plays a key role in the maintenance of peripheral T-cell tolerance^1^ but also restrains anti-viral and anti-tumor T-cell responses^2–4^. PD-1 blockade has been used in the clinic to induce anti-tumor immunity, leading to durable responses in a fraction of patients although most patients respond only transiently^5,6^. This outcome emphasizes the need for better understanding of the molecular mechanisms by which PD-1 mediates its T-cell inhibitory function.

The cytoplasmic tail of PD-1 contains two tyrosine-based structural motifs, an immunoreceptor tyrosine-based inhibitory motif (ITIM) (V/L/I/XpYXX/L/V) and an immunoreceptor tyrosine-based switch motif (ITSM) (TXpYXXV/I)^7,8^. Mutational studies have shown that PD-1 inhibitory function depends on the ITSM phosphotyrosine, which preferentially recruits SHP-2 phosphatase, resulting in downregulation of downstream signaling^9–11^. Mass spectrometry studies have shown that PD-1 phospho-ITSM peptide can act as a docking site in vitro for both SHP-2 and SHP-1, while PD-1 phospho-ITIM peptide can associate only with SHP-2^12^. Although in these in vitro systems both SHP-1 and SHP-2 were found to interact with PD-1 phospho-ITSM^10,12^, live-cell imaging showed that only SHP-2 interacts with PD-1 in live cells^11^. Notably, the ability of PD-1 to exert its inhibitory effects requires simultaneous TCR-mediated stimulation^13–15^, suggesting that T-cell activation signals are necessary for PD-1 to manifest its inhibitory function.

SHP-2 has two tandem SH2 domains, N-terminal (N-SH2) and C-terminal SH2 (C-SH2), followed by a single phosphatase (PTP) domain, and a C-terminal hydrophobic tail with two tyrosine phosphorylation sites (Supplementary Fig. 1a). Extracellular stimuli trigger the binding of SHP-2 via its SH2 domains to tyrosine-phosphorylated receptors for growth factors such as platelet-derived growth factor (PDGF), as well as to tyrosine-phosphorylated docking proteins including insulin receptor substrates (IRSs), signal regulatory protein α (SIRPα; also known as SHP substrate-1 ([SHPS-1]), Grb2-associated binder proteins (Gabs), and fibroblast growth factor receptor substrate (FRS)^16–22^. Such interactions not only activate phosphatase activity but also recruit SHP-2 to sites near the plasma membrane where potential substrate proteins may be located^23–25^. The mechanisms that regulate SHP-2 activity have been extensively studied. At the basal state, the N-SH2 domain of SHP-2 binds the phosphatase domain in an auto-inhibitory closed conformation and directly blocks the active phosphatase site. Interaction of the N-SH2 domain with phosphotyrosine peptide disrupts the interaction of N-SH2 with the phosphatase active site and activates the enzyme (Supplementary Fig. 1b). The C-SH2 domain contributes binding energy and specificity but does not have a direct role in enzymatic activation^24,26^. The crystal structure of SHP-2 has shown that the two SH2 domains of SHP-2 have a roughly antiparallel or perpendicular orientation relative to one another, with the phosphopeptide binding sites lying fully exposed on the surface of the molecule and widely spaced (Supplementary Fig. 1c)^26,27^. Binding of both SH2 domains is required for SHP-2 enzymatic activation^28^.

We examined the mechanism of PD-1: SHP-2 interaction and found that after PD-1 phosphorylation by TCR-proximal Src family tyrosine kinases, SHP-2 can bridge two phosphorylated ITSM-Y248 residues via its N-SH2 and C-SH2 domains, to form a PD-1 dimer. Mutagenesis of the active site of either SH2 domain abrogated SHP-2: PD-1 interaction. The ability of SHP-2 to interact with two ITSM-pY248 residues via its SH2 domains was biophysically documented by isothermal titration calorimetry. Moreover, interaction of SHP-2 SH2 domains with two PD-1 ITSM-pY248 phosphopeptides induced robust enzymatic activation of SHP-2. In T cells, mutagenesis of ITIM-Y248 but not ITIM-Y223 abrogated PD-1-mediated inhibition of interleukin (IL)-2 production. Our results unravel an unexpected mechanism of PD-1: SHP-2 interaction that depends only on ITSM-Y248 and resolve a long-standing conundrum of how a single docking site within the PD-1 cytoplasmic tail is able to activate SHP-2 and PD-1-mediated inhibitory function.

## Results

### Phosphorylation of PD-1 ITSM Y248 by TCR proximal Src family kinases is sufficient for interaction with SHP-2

**Fig. 1:**
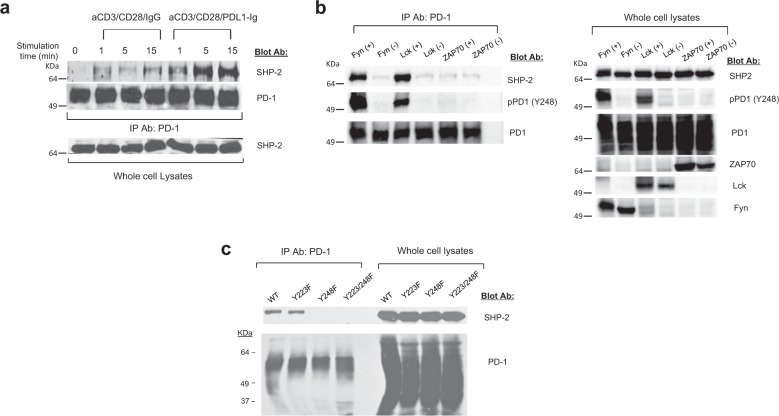
Phosphorylation of PD-1 ITSM Y248 by TCR proximal Src family kinases Fyn and Lck, but not ZAP-70 is required for interaction with SHP-2. **a** Jurkat-PD1 cells were left unstimulated or stimulated with aCD3/CD28/IgG or aCD3/CD28/PDL1-Ig beads for the indicated times, lysates were prepared followed by immunoprecipitation with anti-PD-1 antibody, SDS-PAGE, and western blot with antibodies for SHP-2 and PD-1. SHP-2 expression in whole-cell lysates was examined. **b** COS cells were co-transfected with PD-1, SHP-2, and either the kinase active (+) or inactive (–) form of either Fyn, Lck, or ZAP-70. After PD-1 immunoprecipitation of cell lysates and SDS-PAGE, immunoblot was performed with antibodies for SHP-2, PD-1, or an antibody specific for phosphorylated tyrosine of PD-1 ITSM Y248. Expression of the indicated proteins in the same whole-cell lysates was assessed. **c** COS cells were co-transfected with SHP-2, Fyn kinase active and either PD-1 wild type, PD-1 Y223F, PD-1 Y248F, or PD-1 Y223F/Y248F. PD-1 immunoprecipitation was performed in cell lysates followed by SDS-PAGE and immunoblot with the indicated antibodies. Expression of the same proteins in the same whole-cell lysates was assessed. Results are representative of five independent experiments.

To examine the mechanism of PD-1: SHP-2 interaction and SHP-2 enzymatic activation, we generated Jurkat T cells stably expressing human PD-1 (J-PD1 cells) (Supplementary Fig. 2a). Incubation of J-PD-1 cells with magnetic beads conjugated with αCD3/αCD28/IgG or αCD3/αCD28/PDL1-Ig^1,29,30^ showed that PD-1: SHP-2 interaction was induced by CD3 ligation with or without CD28 costimulation and was enhanced upon simultaneous PD-1 co-ligation (Fig. 1a, Supplementary Fig. 3a, and Supplementary Fig. 4).

To study the role of PD-1 ITSM Y248 phosphorylation, we generated a phospho-ITSM pY248-specific antibody pPD1(Y248)^31^. Since PD-1 is phosphorylated by TCR proximal family kinases^13^, we co-transfected COS cells with PD-1, SHP-2, and either kinase-active or dominant-negative forms of Fyn, Lck or ZAP-70 complementary DNAs (cDNAs)^32–35^. Src family kinases Fyn and Lck, but not ZAP-70, mediated phosphorylation of PD-1 ITSM Y248, and PD-1: SHP-2 interaction (Fig. 1b and Supplementary Fig. 3b). PD-1 Y248 phosphorylation was necessary and sufficient for interaction with SHP-2 because mutation of ITIM (Y223F) did not affect this interaction (Fig. 1c and Supplementary Fig. 3c). In contrast, mutation of ITSM (Y248F) and the double mutation Y223F/Y248F abrogated SHP-2 co-precipitation (Fig. 1c and Supplementary Fig. 3c). Using T cells from mice deficient for Fyn, the TCR most proximal Src family kinase, we found that although PD-1 expression was induced after stimulation, PD-1 phosphorylation was impaired (Supplementary Fig. 5), indicating that Fyn has a physiologic role in inducing PD-1 phosphorylation.

### Both SH2 domains of SHP-2 can mediate SHP-2 binding to PD-1

**Fig. 2:**
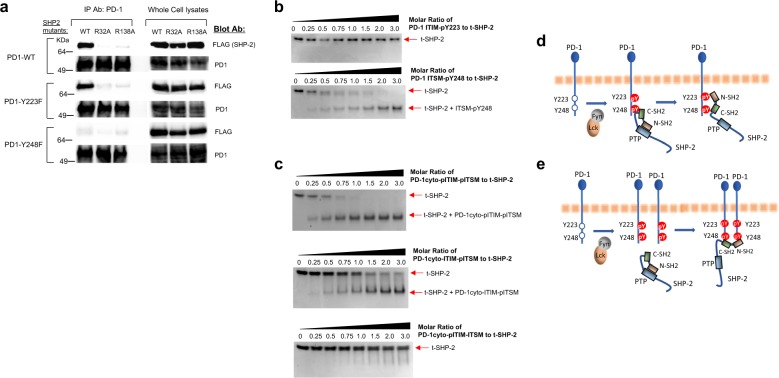
Interaction of both SH2 domains of SHP-2 is required for SHP-2 binding to PD-1. **a** COS cells were transfected with kinase active Fyn, PD-1 WT and either SHP-2-WT, SHP-2-R32A, or SHP-2-R138A mutants FLAG-tagged; Fyn, PD-1-Y223F and either SHP-2-WT, SHP-2-R32A, or SHP-2-R138A mutants FLAG-tagged; or Fyn, PD-1-Y248F and either SHP-2-WT, SHP-2-R32A, or SHP-2-R138A mutants FLAG-tagged. Immunoprecipitation of cell lysates was performed with anti-PD-1 antibody followed by SDS-PAGE and immunoblot with FLAG- or PD-1-specific antibodies. Expression of the indicated proteins in the same whole-cell lysates was assessed. Results are representative of four separate experiments. **b** ITSM-pY248 but not ITIM-pY223 phosphopeptide binds with SHP-2 SH2 domains. For assessment of ITIM-pY223 and ITSM-pY248 binding to SHP-2 SH2 domains, SHP-2 amino acids 1–225, which only contains the tandem N-SH2 and C-SH2 domains in their natural sequence (referred to as t-SHP-2), was used. 2 µM of t-SHP-2 were mixed with either ITIM-pY223 or ITSM-pY248 phosphopeptides at the indicated molar ratios, incubated for 1 h at room temperature and binding was examined by Native PAGE followed by Coomassie blue staining. **c** t-SHP-2 was mixed with a phosphopeptide corresponding to the native sequence of PD-1 cytoplasmic tail in which either both ITIM-Y223 and ITSM-Y248, only the ITIM-Y223, or only the ITSM-Y248 tyrosines were phosphorylated (PD-1cyto-pITIM-pITSM, PD-1cyto-pITIM-ITSM, and PD-1cyto-ITIM-pITSM, respectively).  2μM of t-SHP-2 were mixed with each of the phosphopeptides at the indicated molar ratios, incubated for 1 h hour at room temperature and binding was examined by Native PAGE followed by Coomassie blue staining. **d**, **e** Tentative models of PD-1: SHP-2 interaction after PD-1 phosphorylation.

To determine whether a selective interaction between one of the SHP-2 SH2 domains and a specific PD-1 phosphotyrosine might occur, we transfected COS cells with SHP-2 wild type or SHP-2 with inactivating mutations in the active site of each SH2 domain, specifically arginine 32 residue in the N-SH2 domain and arginine 138 in the C-SH2 domain, which are critical for mediating the binding of each SH2 domain of SHP-2 with phosphotyrosine^36^. Mutagenesis of arginine to alanine at the active site of either SH2 domain (R32A or R138A) nearly abrogated SHP-2 interaction with PD-1 wild type (Fig. 2a and Supplementary Fig. 6a). Similarly, mutagenesis of either SH2 domain equally disrupted the interaction of SHP-2 with endogenous PD-1 in primary human T cells (Supplementary Fig. 6b, c). Wild-type SHP-2 was still able to interact with PD-1 ITIM-Y223F mutant containing intact Y248 but the inactivating mutations of each SH2 domain (R32A and R138A) equally compromised the interaction of SHP-2 (Fig. 2a and Supplementary Fig. 6a). PD-1 ITSM-Y248F mutant disrupted the interaction of PD-1 with wild-type SHP-2 and each of the SH2 domain mutants (Fig. 2a and Supplementary Fig. 6a). Altogether these studies provide evidence that both SH2 domains of SHP-2 are critical for its interaction with PD-1 and that PD-1 Y248 is indispensable for this interaction.

To determine whether SHP-2 SH2 domains display a binding preference for PD-1 ITIM-pY223 or ITSM-pY248, we assessed binding of ITIM-pY223 or ITSM-pY248 phosphopeptide to SHP-2 SH2 domains by native PAGE binding assay. As a consequence of ITSM-pY248 phosphopeptide binding, a truncated SHP-2 protein that contains only the two tandem SH2 domains (t-SHP-2) manifested a shift in electrophoretic ability that was not observed on its incubation with ITIM-pY223 phosphopeptide (Fig. 2b). Thus, when ITIM-pY223 and ITSM-pY248 phosphopeptides are incubated with SHP-2 SH2 domains at the same molar ratio, ITSM-pY248 is the preferred binding partner of SHP-2. Consistently, compared with a peptide containing both phosphorylated tyrosines of the native PD-1 cytoplasmic tail (Fig. 2c, top panel), electrophoretic mobility shift of t-SHP-2 was preserved by a peptide containing phosphorylation of Y248 (PD-1cyto-ITIM-pITSM) but not by a peptide containing phosphorylation of Y223 (PD-1cyto-pITIM-ITSM) (Fig. 2c).

Altogether, these findings are consistent with two tentative models of PD-1: SHP-2 interaction when co-expressed in cells. One possible model is that PD-1 ITSM-pY248 serves as the high-affinity binding site for one of the SHP-2 SH2 domains thereby being a pre-requisite for the binding of the second SH2 domain on PD-1 ITIM-Y223 that serves the low-affinity interaction site (Fig. 2d). This model can provide an explanation why PD-1 ITSM-Y248F disrupted the interaction of PD-1 with either SHP-2 WT or each SHP-2 SH2 active site mutant (Fig. 2a) and why ITIM-Y223F did not affect interaction with SHP-2 WT, but cannot explain why in cells expressing ITIM-Y223F, interaction of PD-1 with each SH2 active site mutant was disrupted (Fig. 2a). A second possible model is that both SH2 domains interact with ITSM-pY248 and because the PD-1 molecule has only one ITSM-Y248, SHP-2 binds phosphorylated ITSM-pY248 residues in two PD-1 molecules using its N-SH2 domain for one PD-1 and its C-SH2 domain for a second PD-1 to form a PD-1 dimer (Fig. 2e). The latter can explain why PD-1 ITSM-Y248F disrupted the interaction of PD-1 with either SHP-2 WT or each SHP-2 SH2 active site mutant (Fig. 2a, bottom panel). This model can also explain why in cells expressing PD-1 ITIM-Y223F the interaction of PD-1 with SHP-2 WT was not affected but the interaction of PD-1 with each SH2 active site mutant was disrupted (Fig. 2a).

### Surface plasmon resonance (SPR) and isothermal titration calorimetry (ITC) analysis of SHP-2 interaction with PD-1

**Fig. 3:**
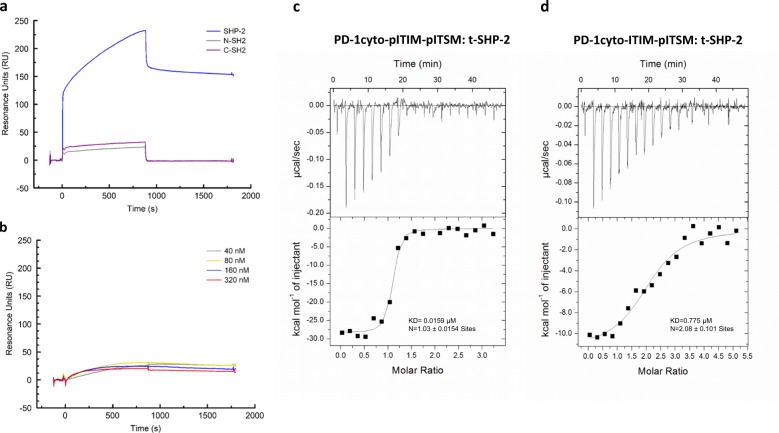
Surface plasmon resonance (SPR) and isothermal titration calorimetry analysis of SHP-2 interaction with PD-1. **a** PD-1 phosphotyrosyl ITSM-Y248 peptide was immobilized on a CM5 BIAcore chip and binding of SHP-2 full-length SHP-2-N-SH2, and SHP-2-C-SH2, all at 320 nM, was determined by SPR. Full binding curves are presented in Supplementary Fig.  8. Sensograms of 900 s (15 min) association and 900 s dissociation time were analyzed and *K*_D_ values were calculated. **b** PD-1 phosphotyrosyl ITIM-Y223 peptide was immobilized on a CM5 BIAcore chip and binding of SHP-2-full-length at the indicated concentrations, was determined by SPR. Results are representative of three experiments. **c**, **d** Isothermal titration calorimetry (ITC) experiments were performed on a MicroCal ITC200 instrument. PD-1 phosphopeptides (PD-1cyto-pITIM-pITSM and PD-1cyto-ITIM-pITSM) and t-SHP-2 protein were prepared in buffer containing 50 mM HEPES pH 7.4, 100 mM NaCl, 5 mM TCEP and stoichiometry of bisphosphorylated PD-1 peptide: t-SHP-2 interaction was assessed as described in Methods.

We used surface plasmon resonance (SPR) to analyze the interaction of purified SHP-2 protein (Supplementary Fig. 7) with immobilized PD-1 ITSM-pY248 and PD-1 ITIM-pY223 phosphopeptides. The anti-phosphotyrosine antibody 4G10 bound with comparable affinity to the immobilized PD-1 ITSM-pY248 and PD-1 ITIM-pY223 phosphopeptides (*K*_D_ = 1.25 nM and *K*_D_ = 2.24 nM, respectively). SHP-2-full-length interacted with PD-1 ITSM-pY248 coated surface in a dose-dependent manner (*K*_D_ = 0.0197 μM) (Fig. 3a and Supplementary Fig. 8a). Under identical conditions, no specific interaction of SHP-2 protein was detected with PD-1 ITIM-pY223-coated surface (Fig. 3b), consistent with our cellular and biochemical results (Fig. 2a, b) showing that ITIM-pY223 was not the preferred interaction site for SHP-2. We generated recombinant proteins of each single SHP-2 SH2 domain (Supplementary Fig. 7) and assessed binding to PD-1 ITSM-pY248. We found that both SH2 domains of SHP-2 are involved in the interaction with PD-1 ITSM-pY248 (Fig. 3a) but the calculated affinities by steady-state analysis of the N-SH2 domain and that of the C-SH2 domain are comparable (1.316 µM vs.1.579 µM, respectively; Supplementary Fig. 8c–f). Importantly, the affinity of each individual SH2 domain is negligible compared with the calculated affinity of SHP-2-full-length (*K*_D_ = 0.0197 μM; Supplementary Fig. 8a). These findings suggest that interaction of both SH2 domains of SHP-2-full-length protein to PD-1 ITSM-pY248 might be responsible for the higher binding of SHP-2-full-length compared with the binding of each individual SH2 domain.

To examine further the nature of PD-1: SHP-2 interaction, we assessed stoichiometry by isothermal titration calorimetry (ITC). We used SHP-2 protein that contains only the two tandem SH2 domains (t-SHP-2) and a phosphopeptide corresponding to the native PD-1 cytoplasmic tail in which both ITIM-Y223 and ITSM-Y248 tyrosines were phosphorylated (PD-1cyto-pITIM-pITSM). In addition, we used a phosphopeptide corresponding to the native PD-1 cytoplasmic tail in which ITSM-Y248 but not ITIM-Y223 was phosphorylated (PD-1cyto-ITIM-pITSM), and a phosphopeptide in which ITIM-Y223 but not ITSM-Y248 was phosphorylated (PD-1cyto-pITIM-ITSM). PD-1cyto-pITIM-pITSM: t-SHP-2 interaction occurred at 1:1 stoichiometry (Fig. 3c). In contrast, PD-1cyto-ITIM-pITSM: t-SHP-2 interaction occurred at 2:1 stoichiometry (Fig. 3d) providing biophysical evidence that the two SHP-2 SH2 domains can interact with ITSM-pY248 from two PD-1 molecules to form a PD-1 dimer. PD-1cyto-pITIM-ITSM: t-SHP-2 interaction was not detectable by ITC indicating that SHP-2-mediated bridging of two PD-1 molecules can be induced by pITSM-Y248 but not pITIM Y223. The affinity of t-SHP-2: PD-1cyto-pITIM-pITSM interaction was higher than the affinity of t-SHP-2: PD-1cyto-ITIM-pITSM interaction (0.0159 µM and 0.775 µM, respectively; Fig. 3c, d), indicating that in the presence of pITSM-Y248, pITIM-Y223 contributes to SHP-2 binding. However, pITSM Y248 can also serve as the sole site for interaction of both SHP-2 SH2 domains, albeit with lower affinity. These results show that when purified proteins are used in a cell-free system, PD-1: SHP-2 interaction can occur in two different ways: (1) SHP-2 SH2 domains can bind one PD-1 molecule likely using both phosphotyrosines, each of which interacts with one SHP-2 SH2 domain; (2) SHP-2 SH2 domains can interact solely with pITSM-Y248 phosphotyrosine on two PD-1 molecules.

### SHP-2 bridges two PD-1 molecules via PD-1 ITSM pY248 in live cells

**Fig. 4:**
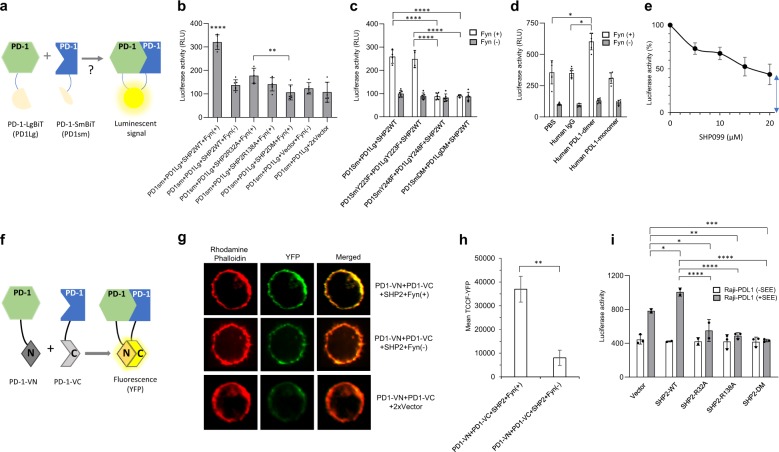
SHP-2 bridges two PD-1 molecules via PD-1 ITSM pY248. **a** The NanoBiT proximity assay: PD-1-LgBiT and PD-1-SmBiT induce luciferase activity only if two PD-1 molecules stably interact resulting in the formation of an active luciferase enzyme. **b** HEK-293 cells were co-transfected as indicated and luciferase activity was assessed. Comparisons were performed by two-way ANOVA and Tukey’s multiple comparisons test (****P < 0.0001, PD1sm + PD1Lg + SHP2WT + Fyn(+) vs. all other samples, **P < 0.005, PD1sm + PD1Lg + SHP2R32A + Fyn(+) vs. PD1sm + PD1Lg + SHP2DM + Fyn(+), *n* = 6 experiments). **c** HEK-293 cells were co-transfected with the indicated constructs together with either kinase active (+) or inactive (-) Fyn and luciferase activity was assessed (****P < 0.0001, *n* = 3 experiments). **d** HEK-293 cells co-transfected with PD1SmBiT, PD1LgBiT and SHP-2 WT, together with either kinase active (+) or inactive (−) Fyn, were cultured in the presence of hPD-L1-Ig (dimer), hPD-L1 monomer or control IgG and luciferase activity was assessed (*P < 0.05, *n* = 3 experiments). **e** HEK-293 cells co-transfected with PD1SmBiT, PD1LgBiT, SHP-2 WT, and kinase active Fyn were cultured in the presence of increasing amounts of the allosteric SHP-2 inhibitor SHP099 or vehicle control (0) and luciferase activity was assessed (arrow indicates baseline luciferase activity). **f** The BiFC assay is based on structural complementation of two non-fluorescent N- and C-terminal fragments of Venus YFP-fluorescent protein fused to a pair of interacting proteins. **g** HEK-293 cells transfected with indicated constructs were analyzed by confocal microscopy. Representative fluorescent images of each indicated transfection condition. Polymerized actin was visualized with rhodamine-conjugated phalloidin. YFP signal from PD1-VN + PD1-VC complementation was visualized in the green channel. Intensity of yellow color in the merged images is indicative of co-localization of PD-1 dimer with the actin cytoskeleton. **h** Quantitative analysis of images from **g** and comparison of total corrected cellular YFP fluorescence (TCCF-YFP) in the indicated conditions after subtraction of TCCF-YFP from control transfection PD1-VN + PD1-VC + 2xVector, **P < 0.005, *n* = 50 from one of two independent experiments. **i** Primary human T cells co-transfected with PD1SmBiT, PD1LgBiT and the indicated SHP-2 constructs were cultured with Raji-PD-L1 with or without prior loading with SEE and luciferase activity was assessed 6 h later (****P < 0.0001, ***P < 0.0005, **P < 0.005, *P < 0.05, *n* = 3 experiments).

Next, we examined whether SHP-2-dependent PD-1 dimer is detected in live cells. We used the NanoBiT proximity assay (Promega), which permits detection of protein: protein interaction in living cells by employing a split luciferase enzyme. To examine whether two PD-1 molecules on the same cell surface are brought into proximity by SHP-2, we generated a pair of PD-1 NanoBiT plasmids: PD-1-LgBiT (PD1Lg), in which the PD-1 cytoplasmic domain was linked to the LgBiT peptide sequence, and PD-1-SmBiT (PD1sm), in which the PD-1 cytoplasmic domain was linked to the SmBiT peptide sequence. As luminescence is generated only when the LgBiT and SmBiT peptides come together to form an active luciferase enzyme^37^, this would be achieved only if two PD-1 molecules stably interact (Fig. 4a).

We transfected HEK-293 cells with PD-1-LgBiT and PD-1-SmBiT, together with SHP-2 and Fyn kinase, to recapitulate TCR-mediated PD-1 phosphorylation and interaction with SHP-2. Assessment of complex formation between PD-1-SmBiT and PD-1-LgBiT by measuring luminescent signal showed that in the presence of kinase-active Fyn but not kinase-inactive Fyn, SHP-2 induced PD-1: PD-1 interaction (Fig. 4b and Supplementary Fig. 9). Expression of PD-1 was confirmed by flow cytometry (Supplementary Fig. 10a). Co-transfection of PD-1-SmBiT and PD-1-LgBiT with kinase active Fyn, together with either SHP-2-R32A or SHP-2-R138A, showed that loss of the active site of either SH2 domain abrogated PD-1: PD-1 interaction (Fig. 4b). Loss of the active sites of both SH2 domains in SHP-2-R32A/R138A double mutant (DM) induced the greatest decline. Mutation of the PD-1 ITSM-Y248 abrogated SHP-2-mediated PD-1: PD-1 interaction, whereas mutation of the PD-1 ITSM-Y223 had no effect (Fig. 4c). Notably, ligation of PD-1 with dimeric recombinant PD-L1 but not monomeric PD-L1 induced a significant increase of PD-1 dimerization and this effect was abrogated in the presence of kinase-inactive Fyn (Fig. 4d).

To further investigate the role of SHP-2 SH2 domains in the formation of the PD-1: PD-1 dimer, we used the allosteric SHP-2 inhibitor SHP099, which prevents the conformational change of SHP-2 and the release of the N-SH2 domain from the PTP site^38^. Addition of increasing amounts of SHP099, but not vehicle control, in HEK-293 cells transfected with PD-1-SmBiT and PD-1-LgBiT, together with active Fyn and SHP-2, showed that preventing the conformational change of SHP-2 SH2 domains induced a dose-dependent inhibition of PD-1: PD-1 interaction (Fig. 4e). Thus, a PD-1: PD-1 complex can be formed in live cells and requires PD-1 phosphorylation and interaction with SH2 domains of SHP-2.

To visualize PD-1 localization and dimer formation we used a Venus-based biomolecular fluorescence complementation (BiFC) assay^39^, based on the structural complementation of two non-fluorescent N- and C-terminal fragments of Venus YFP-fluorescent protein fused to a pair of interacting proteins (Fig. 4f). HEK-293 cells were transfected with PD-1-Venus N-terminus (PD1-VN) and PD-1-Venus-C-terminus (PD1-VC), together with SHP-2 and either kinase active or kinase inactive Fyn, and were analyzed by confocal microscopy^40^. As shown in Fig. 4g, the YFP signal, generated from PD1-VN + PD1-VC complementation, was predominantly derived from the plasma membrane as determined by signal co-localization with the cortical actin cytoskeleton that is adjacent to the plasma membrane. Quantitative analysis showed that total corrected cellular fluorescence at the YFP channel (TCCF-YFP) of cells transfected with PD1-VN, PD1-VC, SHP-2 and active Fyn was significantly higher compared to that of cells transfected with PD1-VN, PD1-VC, SHP-2 and inactive Fyn (Fig. 4h) while total PD-1 expression level was comparable (Supplementary Fig. 10b). These results indicate that SHP-2 can bridge PD-1 molecules localized at the plasma membrane under conditions that promote PD-1 phosphorylation.

To examine whether PD-1 dimers bridged by SHP-2 are formed in T cells, we transfected primary human T cells with PD-1-LgBiT and PD-1-SmBiT. Expression of PD-1 was confirmed by flow cytometry and was comparable to that observed in activated primary human T cells (Supplementary Fig. 10c–e). We used an established cellular model for assessment of PD-1 inhibitory function^13^ and stimulated the transfected primary T cells with Raji cells stably expressing human PD-L1 (Supplementary Fig. 11) with or without prior loading with SEE^40,41^. Co-culture with Raji-PD-L1 loaded with SEE but not without SEE resulted in an elevated luminescent signal that was increased by transfection with SHP-2 WT (Fig. 4i). In contrast, the luminescence signal was significantly diminished by expression of SHP-2-R32A or SHP-2-R138A or the SHP-2-DM that was mutagenized in the active sites of both SH2 domains (Fig. 4i), indicating that these SHP-2 mutants functioned as dominant negative by competing with endogenous SHP-2 for interaction with PD-1. Thus, similarly to the pathway complementation approach employed in HEK-293 cells, PD-1: PD-1 interaction is also mediated by the SH2 domains of SHP-2 in primary T cells.

### Interaction of SHP-2 with two PD-1 ITSM-pY248 residues induces SHP-2 phosphatase activation

SHP-2 activation requires binding of both SH2 domains for appropriate conformational changes that lead to exposure of the catalytic site^17,18,24,26,27,42^. Our studies indicated that two possible modes of PD-1: SHP-2 interaction can occur in a cell-free system, one that involves binding of PD-1 ITIM-pY223-ITSM-pY248 with the tandem SH2 domains of SHP-2, and a second that involves binding of ITSM-pY248 from two PD-1 molecules on the two SH2 domains of SHP-2 (Fig. 3c, d). For these reasons, we examined whether interaction of SHP-2 with phosphorylated PD-1 Y223 and Y248 tyrosines or with two phosphorylated Y248 tyrosines would differentially activate SHP-2 phosphatase.

We used the previously established monophosphorylated peptide IRS-1-pY1172 (pIRSY1172) that induces low-level activation of SHP-2, the IRS-1-pY727 (pIRSY727) as a negative control^42^, monophosphorylated PD-1 ITIM-pY223 (pITIM) and ITSM-pY248 (pITSM) peptides, and bisphosphorylated peptides containing various combinations of PD-1 pITIM and PD-1 pITSM that we designed to match the spacing between the phosphopeptide binding sites of the SHP-2 SH2 domains^27^. The distance between the phosphopeptide binding sites of the SH2 domains is approximately 50 Å in the crystal structure of the tandem SH2 domains and is critical for phosphoprotein recognition and enzymatic activation of SHP-2^26,27^. We generated bisphosphorylated peptides that match this spacing requirement, by covalent joining either one pITIM and one pITSM, two pITIM or two pITSM with a 4 amino hexanoic acid (Ahx) spacer^27^ (bisphosphoryl ITIM-ITSM: pITIM-pITSM: bisphosphoryl ITIM-ITIM: bpITIM; and bisphosphoryl ITSM-ITSM: bpITSM) to examine whether interaction of SHP-2 with one pITIM and one pITSM, two pITIM or two pITSM might activate SHP-2 phosphatase activity.

**Fig. 5:**
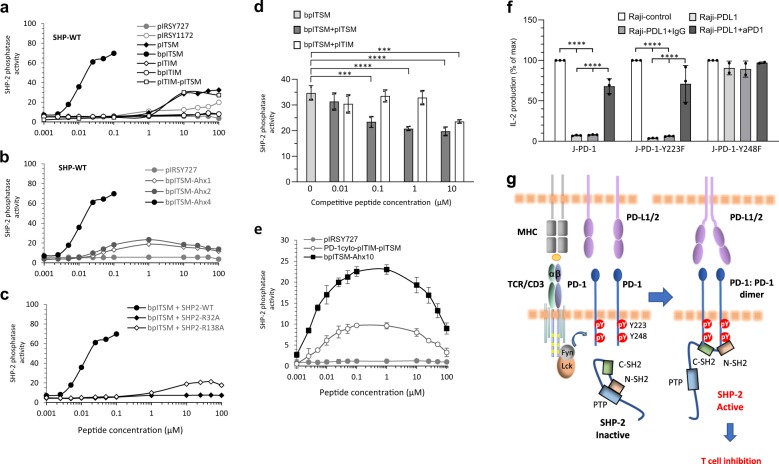
Interaction of SHP-2 SH2 domains with two PD-1 ITSM pY248 residues is required for activation of SHP-2 phosphatase activity. **a** SHP-2-WT was incubated with 20 µM DiFMUP as substrate in the presence of the indicated phosphopeptides and phosphatase activity was assessed as described in Methods. **b** Phosphatase activity of SHP-2-WT incubated with bpITSM phosphotyrosyl peptides containing a 4-, 2-, or 1-Ahx spacer; pIRSY727 was used as negative control. **c** Phosphatase activity of SHP-2-WT, SHP-2-R32A, or SHP-2-R138A incubated with the indicated concentrations of bpITSM phosphotyrosyl peptide containing a 4-Ahx spacer. **d** Phosphatase activity of SHP-2-WT in the presence of phosphotyrosyl bpITSM peptide (0.01 µM) either alone or with increasing amounts of monophosphorylated pITIM or monophosphorylated pITSM peptide. Results are representative of three independent experiments. **e** Phosphatase activity of SHP-2-WT in the presence of a phosphopeptide corresponding to the native sequence of PD-1 cytoplasmic tail in which both ITIM-Y223 and ITSM-Y248 tyrosines were phosphorylated (PD-1cyto-pITIM-pITSM) or with a bpITSM phosphotyrosyl peptide containing a 10-Ahx spacer. pIRSY727 was used as negative control. In panels **a**–**e** results from one representative of 3–10 independent experiments are shown. **f** Jurkat T cells stably expressing PD-1-WT (J-PD-1), PD-1-Y223F (J-PD-1-Y223F), or PD-1-Y248F (J-PD-1-Y248F) were co-cultured with Raji-control or Raji-PD-L1 cells loaded with SEE. Where indicated, anti-PD-1 blocking antibody or isotype control was added in the cultures. Culture supernatants were collected at 24 h and IL-2 production was measured. Results are expressed as % of maximum IL-2 production induced in each cell line by Raji-control cells loaded with SEE (****P < 0.0001, *n* = 3 experiments). **g** Model for PD-1: PD-1 bridging and activation of SHP-2 phosphatase activity: An “inside-out” regulation of PD-1: PD-1 dimer complex formation is initiated by TCR-mediated activation of TCR proximal Src family kinases, which induce phosphorylation of tyrosines in PD-1 cytoplasmic tail. The stabilization provided by PD-1 ligands allows the appropriate proximity of two phosphorylated PD-1 molecules to induce binding of both SH2 domains of SHP-2 to ITSM-pY248 and activation of SHP-2, leading to inhibition of T-cell responses.

Measurements of SHP-2 phosphatase activity using DiFMUP (6,8-difluoro-4-methylumbelliferyl phosphate) substrate showed that pITIM and bpITIM peptides did not induce an increase in the catalytic activity of SHP-2 compared to the established negative control pIRSY727. pITSM induced an increase in the catalytic activity of SHP-2 compared to pIRSY727 and pIRSY1172 phosphopeptides, but only at concentrations above 1 µM, and similar results were observed with pITIM-pITSM bisphosphorylated peptide (Fig. 5a). Strikingly, bpITSM caused a robust increase in SHP-2 activity at 1000-fold lower phosphopeptide concentrations than pITIM-pITSM (Fig. 5a). Progressive shortening of the linker length led to a pronounced drop-off in catalytic activity induced by bpITSM (Fig. 5b), confirming that this phosphopeptide can activate SHP-2 phosphatase only when engaged under conditions that meet the spatial requirements of the tandem SH2 domains of SHP-2. Compared with bpIRS, a known bisphosphoryl peptide activator of SHP-2 catalytic activity^27,38,42^, PD-1 bpITSM was approximately three times more potent in inducing SHP-2 activation (Supplementary Fig. 12). Loss of the active site of either N-SH2 or C-SH2 domain in SHP-2-R32A and SHP-2-R138A, respectively, abrogated SHP-2 activity caused by bpITSM (Fig. 5c). A small induction of SHP-2 activity in the SHP-2-R138A was observed at peptide concentrations above 1 µM (Fig. 5c). Most likely, at these peptide concentrations, high-affinity binding of the bpITSM to the intact N-SH2 domain of SHP-2-R138A mediates some degree of SHP-2 activation. Thus, low-level SHP-2 activation can be induced by binding with two optimally spaced PD-1 ITIM-pY223 and ITSM-pY248 phosphopeptides but significantly higher SHP-2 activity is induced by binding of both SH2 domains with two PD-1 ITSM-pY248 phosphopeptides, spaced in a similar manner. Although in bpITSM the two PD-1 ITSM phosphopeptides are placed in tandem, these results indicate that binding of both SH2 domains on ITSM induces strong enzymatic activation of SHP-2. As PD-1 molecule has only one ITSM, the latter can be achieved in cells by interaction of the two SHP-2 SH2 domains with two optimally spaced PD-1 molecules. This is supported by our biochemical results (Fig. 2a), ITC results (Fig. 3d), and cellular studies showing that SHP-2-dependent PD-1 dimer formation is detected in live cells (Fig. 4).

To further investigate the role of pITIM-Y223 and pITSM-Y248 in the induction of SHP-2 activity, we used bpITSM, phosphopeptide that could induce optimal SHP-2 activation, and supplemented the reaction with increasing amounts of monophosphorylated pITIM or monophosphorylated pITSM peptide to assess if they could compete with bpITSM phosphopeptide for SHP-2 binding and phosphatase activation. Although pITSM decreased SHP-2 phosphatase activity in a dose-dependent manner, pITIM disrupted SHP-2 activation induced by the bpITSM phosphopeptide only when added at very high molar concentrations (1000x excess of bpITSM) (Fig. 5d). These data indicate that SHP-2 binding to ITSM is preferred, and for this reason, pITSM that can only bind one SH2 domain, competes with bpITSM for dual SH2 domain binding and compromises phosphatase activation more effectively than pITIM.

To design conditions that more specifically resemble SHP-2 activation by PD-1 phosphotyrosines, we generated a phosphopeptide corresponding to the native sequence of PD-1 cytoplasmic tail in which both ITIM-Y223 and ITSM-Y248 tyrosines were phosphorylated (PD-1cyto-pITIM-pITSM). To recapitulate binding of SHP-2 with two pITSM-Y248 spaced similarly to the phosphotyrosines of PD-1 cytoplasmic tail, we generated a bisphosphorylated peptide by covalently joining two pITSM-Y248 phosphopeptides with a 10-Ahx spacer (bpITSM-Ahx10), which corresponds to the distance of PD-1 phosphotyrosines. Although PD-1cyto-pITIM-pITSM induced catalytic activity of SHP-2 compared to the negative control pIRSY727, bpITSM-Ahx10 caused a higher increase in SHP-2 activation (Fig. 5e) that was measurable only when using 3x lower enzyme concentrations than in all other phosphatase assays.

Our findings that SHP-2 has the biophysical capacity to interact with two phosphorylated PD-1-pY248 residues identified by ITC (Fig. 3d) and confocal microscopy (Fig. 4g, h) and the ability to undergo activation by binding of its SH2 domains on two PD-1-pY248 phosphopeptides (Fig. 5a–e) reveal a new mechanism of SHP-2: PD-1 interaction and SHP-2 activation, which might account for the previous observations that only pY248 is indispensable for PD-1 inhibitory function in live T cells^9,10^. Mutagenesis of either PD-1 ITIM-pY223 or ITSM-pY248 would equally disrupt PD-1 inhibitory function if both these phosphotyrosines were involved in PD-1-mediated activation of SHP-2 in these cells. The fact that only mutagenesis of PD-1 ITSM-pY248 results in loss of PD-1 inhibitory function indicates that the dominant mechanism of PD-1-mediated SHP-2 activation in T cells involves the interaction of both SHP-2 SH2 domains with two ITSM-pY248. Consistent with this rationale, co-culture with PD-L1-expressing Raji cells loaded with SEE abrogated IL-2 production in J-PD-1 and J-PD-1-Y223F T cells, which express an intact ITSM-Y248, and this inhibitory effect was significantly reversed by PD-1 blocking antibody (Fig. 5f and Supplementary Fig. 13). In contrast, J-PD-1-Y248F T cells, which express an intact ITIM-Y223, but a mutagenized ITSM-Y248 (Supplementary Fig. 2), were resistant to PD-L1-mediated inhibition (Fig. 5f). Altogether, our data favor a model of PD-1: PD-1 dimer complex formation, which is initiated by TCR-mediated activation of kinases that lead to phosphorylation of PD-1-ITSM Y248 and binding of both SHP-2 SH2 domains to form a PD-1 dimer resulting in SHP-2 phosphatase activation and inhibition of activated T-cell responses (Fig. 5g).

## Discussion

Although the inhibitory function of PD-1 has been previously attributed to the interaction of SHP-2 with PD-1 ITSM-pY248^9–11^, it has remained poorly understood how PD-1-mediated SHP-2 activation might be mechanistically regulated. In the present study we determined that PD-1 can induce robust SHP-2 catalytic activity in the presence of two phosphorylated ITSM motifs precisely spaced to meet the distance between the phosphotyrosine binding sites of the SHP-2 SH2 domains. A similarly designed bisphosphorylated peptide generated by covalent joining of one PD-1 ITIM-pY223 and one ITSM-pY248 phosphopeptide could induce SHP-2 catalytic activation only at 1000x higher concentrations and at significantly lower magnitude. The superior ability of the bisphosphorylated bpITSM-Y248 peptide to induce SHP-2 activation in vitro was correlated with PD-1-mediated inhibition of antigen-induced IL-2 production in T cells, which was abrogated when PD-1 ITSM-pY248 but not when ITIM-pY223 was mutagenized to phenylalanine.

Our studies showed that PD-1: SHP-2 interaction and SHP-2 catalytic activation required the presence of two intact SH2 domains in SHP-2 because loss of the active site of either SH2 domain disrupted the interaction with PD-1 and the induction of PD-1-mediated SHP-2 activation. The mandatory role of two precisely spaced PD-1 ITSM-pY248 and the requirement of both intact SH2 domains of SHP-2 for PD-1: SHP-2 interaction and PD-1-mediated SHP-2 activation are consistent with a model in which SHP-2 binds phosphorylated ITSM-Y248 residues in two PD-1 molecules using its N-SH2 domain for one PD-1 and its C-SH2 domain for the second PD-1 to form a PD-1 dimer (Fig. 5g). Indeed, we found that interaction of a PD-1 ITSM-pY248 phosphopeptide with the tandem SH2 domains of SHP-2 occurred in 2:1 stoichiometry. Moreover, after PD-1 phosphorylation by Fyn, PD-1 dimers were detected in live cells, and were disrupted by the loss of the active site of either SHP-2 SH2 domain (Fig. 4b). Such mechanism of interaction of SHP-2 SH2 domains is not unprecedented. Only one (Y1009) of the seven documented phosphorylation sites of the PDGF receptor is found to preferentially interact with SHP-2^16,43^. Tyrosines 1009 and 1021 of the PDGF receptor are spaced similarly to known collinear docking sites for tandem SH2 domains but mutation of Y1021 does not interfere with SHP-2 binding in cells. These results suggested that SHP-2 binds either one Y1009 of a PDGF receptor monomer using only one SH2 domain or two Y1009 residues in an activated PDGF receptor dimer using both SH2 domains. The latter is supported by the structural studies of SHP-2 binding with PDGR receptor^27^ and by the requirement for binding of both SH2 domains for activation of SHP-2 phosphatase activity^28^. Our results are consistent with a similar mechanism of SH2 domain binding by PD-1.

A tentative model for SHP-2 activation by PD-1 would be the interaction of the tandem SH2 domains with the phosphorylated tyrosines in PD-1 ITIM-pY223 and PD-1 ITSM-pY248 similarly to that proposed for IRS^28^. Our studies showed that both PD-1 ITIM-pY223 and ITSM-pY248 could contribute to induce SHP-2 activation in vitro, consistent with previous findings^44^. However, our studies in live cells showed that PD-1-mediated inhibitory function was reversed by mutagenesis of Y248 but not Y223, a finding previously reported by other groups^9–11^. Together with these observations, our results that a bisphosphorylated bpITSM peptide induced strong SHP-2 phosphatase activity, and that SHP-2-dependend PD-1 dimers are detected in T cells during antigen-stimulation and PD-1 ligation, support the conclusion that, in live T cells, SHP-2 is preferentially activated by binding of both SH2 domains on ITSM-pY248 of two properly spaced adjacent PD-1 molecules. It should be noted that although PD-1 pITIM-Y223 does not appear to be a site involved in PD-1-mediated activation of SHP-2 and inhibitory function in the cells, it might have a role in regulating PD-1 function by other mechanisms including altering the charge or conformation of PD-1 cytoplasmic tail or by mediating binding of a yet unidentified protein. The latter might render pITIM-Y223 unavailable for binding with SHP-2, enforcing interaction of SHP-2 SH2 domains on ITSM-pY248 of two different PD-1 molecules and explaining why only pY248 is indispensable for the PD-1 inhibitory function. Such properties of pITIM-Y223 might be selectively promoted under certain physiologic conditions or in distinct subcellular compartments for fine tuning of PD-1 function.

PD-1 and CTLA-4 are inhibitory receptors of the CD28/CTLA-4/B7-1/B7-2 superfamily^45^. CTLA-4 is a covalent dimer^46,47^. Its higher avidity than CD28 for the B7 ligands results from its higher affinity, but also from the binding of each CTLA-4 dimer to two divalent B7 molecules^48^. The crystal structure of CTLA-4: B7 complexes suggests that CTLA-4 covalent dimer can bind to noncovalent dimers of B7-1 to form a lattice of CTLA-4-B7 interactions. Such a lattice can function to form a stable signaling complex at the T cell: APC interface^49,50^. In contrast to CTLA-4, PD-1 does not have the conserved cysteine located proximal to the transmembrane domain, thus, it is not structurally equipped to form a covalent dimer and crystallography studies of the PD-1: PD-L1 binding regions have shown that PD-1 stays as a monomer in solution^51^. Our studies show that PD-1 can form a PD-1: PD-1 noncovalent dimeric complex by a previously unidentified mechanism. Formation of such complex is guided by an “inside-out” signaling sequence induced by TCR-mediated phosphorylation of PD-1. Our results suggest that formation of this PD-1: PD-1 dimer is essential for inhibitory function in live cells because only mutagenesis of the PD-1 phosphotyrosine that is required both for PD-1 dimer formation and SHP-2 enzymatic activation abrogated PD-1-mediated inhibition during antigen-mediated stimulation.

An additional unexpected finding of our studies is that phosphorylation of PD-1 cytoplasmic tail also has an active role in PD-L1-mediated PD-1 oligomerization because PD-1: PD-1 dimer formation induced by dimeric PD-L1 was impaired when phosphorylation of PD-1 cytoplasmic tail was compromised by the expression of a kinase dominant-negative Fyn. Since ITSM-Y248 phosphorylation is required for SHP-2 enzymatic activation, this finding explains why TCR-mediated signaling is required concomitantly with PD-1 ligation by its natural ligands to induce PD-1 inhibitory function. The stabilization provided by PD-1 ligands together with the phosphorylation of PD-1 cytoplasmic tyrosines allows the appropriate proximity of two phosphorylated PD-1 molecules to induce binding of both SH2 domains of SHP-2 on PD-1 ITSM-pY248 and SHP-2 activation, leading to inhibition of T-cell responses. Notably, early studies reported that activation of the Src family kinase Fyn, which we identified here to induce PD-1 phosphorylation and interaction with SHP-2, has an active role in inducing and maintaining the anergic T-cell state^52,53^. It will be important to determine in future studies whether the altered signaling state in anergic and exhausted T cells might favor PD-1: PD-1 complex formation and activation of SHP-2, leading to T-cell immune dysfunction.

As SHP-2 activation has been linked to PD-1 inhibitory function^9–12^ a recent study examined whether mice with T-cell-specific SHP-2 deficiency might be resistant to exhaustion induced by PD-1 in chronic viral infections or display enhanced anti-tumor responses to immunogenic tumors^54^. CD8^+^ T cells from these mice did not show an advantage in controlling chronic viral infections but were rather less polyfunctional than control CD8^+^ cells. These mice displayed comparable improvement of anti-tumor immunity in response to PD-1 blockade. Although these results are intriguing and suggest the presence of additional mediators of PD-1 inhibitory function, this study did not determine whether interaction of PD-1 with other phosphatases such as SHP-1 could play a compensatory role in T-cell inhibitory mechanism in the absence of SHP-2, as recently reported^55^. Importantly, interaction with PD-1 is not the sole function of SHP-2 in T cells. SHP-2 exists in various intracellular pools, interacts with multiple molecular partners and is involved in various functions. For example, SHP-2 is required for TCR-mediated signaling^56^ and for insulin receptor signaling^18,28^, which has a critical role in T-cell responses^57,58^. Because as previously observed^13,14^ and mechanistically dissected by our present studies, the ability of PD-1 to manifest its inhibitory function requires T-cell activation, the relevance of PD-1-mediated inhibition in SHP-2 deficient T cells is unclear.

Similarly to several other signaling molecules that participate in the TCR-activation signaling cascade, PD-1 partitions in the TCR microclusters which consist of TCR and proximal signaling molecules^59,60^. It was previously observed that PD-1 is recruited to the TCR microclusters during antigen recognition and inhibits T-cell activation by bringing SHP-2 to the TCR proximal signaling molecules^11^. Recruitment of PD-1 in the TCR microclusters can occur in the absence of ITSM-Y248 phosphorylation but fails to inhibit T-cell activation^11^. Our present studies suggest that not only ITSM-Y248 phosphorylation but also spatial distribution of phosphorylated PD-1 molecules in proximity to TCR signaling substrates in the TCR microclusters will affect the inhibitory function of PD-1 because only PD-1 molecules bridged through pITSM-Y248 by the two SH2 domains of SHP-2 at such a distance that can induce SHP-2 conformational change allowing activation of SHP-2 phosphatase will be responsible for inhibition of T-cell responses.

Our findings explain why only ITSM-Y248 has an indispensable role in PD-1-mediated inhibitory function and unravel a mechanism of how SHP-2 enzymatic activation can be induced by one PD-1 phosphotyrosine. Our results have implications for the development of new strategies to prevent the PD-1 inhibitory signaling by disrupting PD-1 dimerization.

## Methods

### Cell cultures

Primary human T cells were purified using magnetic beads (Stemcell Technologies, Cambridge, MA) from peripheral blood mononuclear cells (PBMC) of normal healthy volunteer blood donors. A protocol for human PBMC collection had been approved by the Institute’s IRB and ethics committee. Primary mouse T cells were purified from spleens and lymph nodes using magnetic beads (Stemcell Technologies, Cambridge, MA). Cultures of primary human T cells, Jurkat, Raji and COS cells were performed in 37^ o^C/5% CO_2_ incubator in RPMI-1640 supplemented with 2 mM l-glutamine (Cellgro/Mediatech, Manassas, VA), 10% heat-inactivated fetal bovine serum (FBS) (Atlanta Biologicals, Flowery Branch, GA), 10 mM HEPES, 1 mM sodium pyruvate, 50 U/ml Pen/Strep (from Cellgro/Mediatech, Manassas, VA), and 15 µg/ml gentamycin (from Gibco/Invitrogen, Grand Island, NY). Gibco 293H cells (Fisher Scientific) were cultured in Dulbecco's modified Eagle medium (DMEM) supplemented with 10% heat-inactivated FBS, 10 mM HEPES, 1% glutamax, 1% Pen/Strep, 15 µg/ml gentamycin.

Jurkat cells were stably transfected with PD-1 and cultured in presence of 5 µg/ml selection antibiotic blasticidin. Before use in signaling experiments, Jurkat T cells were rested overnight at 37^ o^C in RPMI-1640 containing 2% FBS. Primary human or mouse T cells were rested under the same conditions for 1 h. Raji cells were stably transfected with PD-L1 and cultured in presence of 10 µg/ml blasticidin. For short-term activation, Jurkat, primary human T cells and primary mouse T cells were resuspended at 100 × 10^6 ^cells/ml in RPMI-1640 containing 10 mM HEPES and mixed with an equal volume of RPMI/HEPES containing equal numbers of tosylactivated magnetic beads conjugated with the indicated antibodies or fusion proteins. The mixture was centrifuged for 1 min at 300 × *g* at room temperature and placed immediately at 37 ^o^C for the indicated time points. Preparation of Dynabeads M-450 (Thermo Scientific) tosylactivated magnetic beads using anti-CD3 (UCHT1, Biolegend) and anti-CD28 (CD28.2, Biolegend) mAb was done as previously described^29^. Preparation of Dynabeads coated with anti-mouse antibodies CD3 (clone 145-2C11, Biolegend) and anti-CD28 (clone 37.51, Biolegend) was done by the same method. For Raji-mediated stimulation cells were resuspended at 1 × 10^6 ^cells/ml in RPMI complete medium and loaded with 0.5 ng/ml SEE (Toxin Technologies) by 30 min rotation at 37 ^o^C followed by three washes to remove excess SEE. Jurkat T cells or primary human T cells were cultured in 96-well tissue culture plates, at 10^5^ cells/well with equal numbers of Raji cells (with or without SEE loading) in a final volume of 100 µl. When indicated, a PD-1 blocking antibody (clone EH12) or an isotype control IgG was added in the cultures.

Fyn KO mice (pp59^fyn^ KO; JAX stock #002271)^61^ and wild-type control mice were purchased from The Jackson Laboratory (Bar Harbor, ME). Mice of either sex at 6–8-weeks of age were used. All procedures were in accordance with National Institutes of Health Guidelines for the Care and Use of Animals and a relevant protocol had been approved by the Institutional Animal Care and Use Committee. Activation of mouse T cells was performed with 1 µg/ml a-CD3 (clone 145-2C11, Biolegend) and 1 µg/ml a-CD28 (clone 37.51, Biolegend). For staining of mouse T cells anti-mouse PD-1-PE (clone RMP1-30), anti-mouse CD4-Pacific blue (clone GK1.5) and anti-mouse CD8a-APC (clone 53.6.7) antibodies (Biolegend) were used followed by flow cytometry.

### Cell transfection

For transfection experiments, COS cells were transfected by GeneJuice transfection reagent (EMD Millipore Corp., Billerica, MA) according to the manufacturer’s instructions. Primary human T cells were transfected using the Nucleofector system and human primary transfection kit (Lonza, VPA-1002) according to the manufacturer’s instructions.

### Immunoprecipitation and immunoblotting

Cell lysates were prepared in lysis buffer containing 50 mM Tris-HCl, pH 7.4, 150 mM NaCl, 2 mM MgCl_2_, 10% glycerol and 1% NP-40 supplemented with 2 mM sodium orthovanadate, 1 mM sodium fluoride, 1 mM phenylmethylsulfonyl fluoride (PMSF), and protease Inhibitor Cocktail (Thermo Scientific) and were resolved by sodium dodecyl sulfate–polyacrylamide gel electrophoresis (SDS-PAGE) followed by western blotting with the following antibodies: SHP-2 cat# sc-280, Santa Cruz Biotechnology; Fyn Cat# sc-16, Santa Cruz Biotechnology; Lck Cat# 06-583, EMD Millipore; ZAP-70 (clone 2F3.2), Cat# 05-253, EMD Millipore; FLAG (clone M2) Cat#F3165, Sigma; anti-pY (clone 4G10) Cat# 05-321, EMD Millipore. The mouse monoclonal anti-PD-1 antibodies clones EH12 and EH33 have been previously described^62^. The rabbit polyclonal anti-phospho-Y248 (ITSM) PD-1 antibody was developed in our laboratory^31^. Immunoprecipitations were performed with PD-1 mAb clone EH12 covalently conjugated to Dynabeads protein G (Thermo Scientific). Antibody-coated beads were washed in IP buffer (lysis buffer without NP-40) and subsequently incubated with 500 µg of cell lysates overnight at 4 ^o^C with gentle rotation. After SDS-PAGE, proteins were transferred to a nitrocellulose membrane, followed by western blotting with the indicated antibodies, and images were captured with digital imager FluorChem E (Proteinsimple, San Jose, CA).

### DNA constructs, cloning, and mutagenesis

SHP-2 cDNA (Addgene, Cambridge, MA) was used to generate glutathione-S-transferase (GST) fusions to SHP-2 wild-type full-length (SHP-2-WT-FL) using the pGEX 4T-3 vector (GE Healthcare Life Sciences, Marlborough, MA) and Flag-tagged SHP-2 using the p3xFLAG-CMV10 vector (Addgene, Cambridge, MA). Human PD-1 and PD-L1 cDNAs were expressed in pEF6 vector. For NanoBiT experiments in HEK-293 cells, human PD-1 cDNA was cloned in Large BiT pBit1.1C[TK/LgBit] and in Small BiT pBit2.1C[TK/SmBit] vectors (Promega). For NanoBiT experiments in human T cells PD-1 cDNA was cloned in Large BiT pBiT1.3-C [CMV/LgBiT/Hyg] and in Small BiT pBiT2.3-C [CMV/SmBiT/Blast] vectors (Promega). For confocal microscopy experiments in HEK-293 cells, human PD-1 full-length was cloned in frame with VN173 or VC155 of Venus protein^39^. hPD-1 VN173 and hPD-1 VC155 were synthesized by gBlock (IDT) and each one was cloned into pBit1.1C[TK/LgBit] vector (Promega) after removing LgBiT. For mutagenizing the human PD-1 tyrosine residues Y223 (within the ITIM motif) and Y248 (within the ITSM motif), and the arginine residues of SHP-2 R32 (within the N-SH2 domain) and R138 (within the C-SH2 domain) the QuickChange Lightning Site-Directed Mutagenesis kit from Agilent Technologies was used. All mutations were confirmed by sequencing. For all cloning reactions, the In-Fusion HD cloning kit from Clontech Laboratories, Inc., Mountain View, CA, was used according to the manufacturer’s instructions. The cDNAs for kinase active and kinase inactive forms of Fyn, Lck, and ZAP-70 were previously described^32–35^. cDNA of Fyn and Lck was kindly provided by Dr. Roger Perlmutter (University of Washington, WA) and cDNA for ZAP-70 was provided by Dr. Arthur Weiss (University of San Francisco, CA). Each of these cDNAs was inserted in pcDNA1.1/Amp vector (Invitrogen)^40^.

### NanoBiT assay for receptor dimerization

For experiments with HEK-293 cells, one day before transfection, HEK-293 were plated as 20,000 cells in 0.1 ml DMEM complete medium per well, in six replicates per condition, on a white 96-well tissue culture plate. The following day cells were transfected by Fugene HD (Promega Corporation, Madison, WI) according to the manufacturer’s instructions. A total amount of 100 ng of cDNA was transfected, which included equal amounts (25 ng) of each plasmid for conditions in which four different cDNAs were used. When <4 plasmids were used, the total amount of transfected cDNA was made 100 ng by addition of empty vector. Thirty-hours after transfection, 25 µl of 1X Nano-Glo Live-Cell Assay Substrate (Promega) was added per well and luminescence was read immediately on a SpectraMax M3 plate reader (Molecular Devices). Where indicated, the allosteric SHP-2 inhibitor SHP099 (Medchem Express) was added in the cultures in increasing concentrations; vehicle (DMSO) was used as control. For experiments with primary human T cells a total amount of 2 µg of cDNA was transiently co-transfected using the Nucleofector system as described in the Cell Transfection section. The 2 µg of cDNA included equal amounts (0.67 µg) of each of three different plasmids, PD1LgBiT, PD1SmBiT and either empty vector, SHP-2-WT, SHP-2-R32A, SHP-2-R138A, or the double mutant SHP-2-DM. Cells were co-cultured with Raji-PD-L1 with or without loaded SEE and complex formation between PD-1-SmBiT and PD-1-LgBiT was assessed 6 h later by luciferase assay.

### Biomolecular fluorescence complementation (BiFC) assay for receptor dimerization and localization

HEK-293 cells were plated as 100,000 cells per well on a 24-well tissue culture plate, in three replicates per condition in DMEM complete medium and were transfected by Fugene HD (Promega Corporation, Madison, WI) according to the manufacturer’s instructions. A total amount of 500 ng of cDNA was transfected, which included equal amounts (125 ng) of each plasmid for conditions in which four different cDNAs were used. When <4 plasmids were used, the total amount of transfected cDNA was made 500 ng by addition of empty vector. Thirty-hours after transfection, HEK-293 cells were harvested and prepared for confocal imaging as previously^40^. For confocal microscopy, 0.25 × 10^6^ cells were seeded onto poly-l-lysine–coated slides (Sigma), fixed with 4% (w/v) paraformaldehyde in PHEM buffer [60 mM Pipes, 25 mM Hepes, 10 mM EGTA, 2 mM MgCl_2_, and 120 mM sucrose (pH 7.3)], and permeabilized with 0.1% (v/v) Triton X-100 in Tris-buffered saline. Samples were analyzed with a Perkin Elmer UltraView VoX spinning-disk confocal microscope at the Cellular Imaging Core of Children’s Hospital Boston. Polymerized (F)-actin of the cortical cytoskeleton was visualized with rhodamine-conjugated phalloidin in the red channel and YFP signal from PD1-VN + PD1-VC complementation was visualized in the green channel. A minimum of eight 1-µM z-slices were acquired for each cell and a *z*-stack was created. Processing of images was performed with freeware software paint.net. Quantitative analysis of the total corrected cellular fluorescence (TCCF) = integrated density – (area of selected cell × mean fluorescence of background readings) at the YFP channel was calculated by ImageJ 1.50b software as previously described^63^. Mean value of TCCF-YFP of cells transfected with PD1-VN + PD1-VC + 2xVector was subtracted from the mean TCCF-YFP of cells transfected with PD1-VN + PD1-VC + Fyn(+) or PD1-VN + PD1-VC + Fyn(-). Forty-five cells were analyzed for each condition and the experiment was repeated two times. PD-1 expression was assessed in the same samples by flow cytometry by surface staining with anti-PD-1-APC (clone EH12.EH7, Biolegend) using a Gallios flow cytometer (Beckman Coulter).

### GST-SHP-2 protein production and purification

For preparation of purified recombinant proteins for Biacore surface plasmon resonance analysis, GST-fusion constructs of SHP-2 were expressed in *E. coli* BL21(D3)LysS. One-hundred microliters of bacteria were inoculated in 50 ml LB medium containing 100 µg/ml ampicillin and cultured overnight at 37 ^o^C on a shaker at 225 rpm. The following day cultures were supplemented with 300 ml LB containing ampicillin and growth was continued until OD_600nm_ = 0.6. Protein expression was induced using 200 µM IPTG for 3 h at 37^ o^C. Proteins were extracted by B-PER bacterial protein extraction kit (Thermo Scientific, Rockford, IL) and purified on glutathione-sepharose (GE Healthcare Life Sciences, Marlborough, MA). The GST proteins were captured by incubating 10–15 ml of bacterial lysate (containing the GST-SHP-2 proteins) with 2 ml glutathione-sepharose (1 ml bed volume) for 2 h and gentle rotation at 4^ o^C. Glutathione-sepharose was washed 3 × 10 ml phosphate-buffered saline (PBS) and GST tag was removed by mixing with 50 units thrombin (GE Healthcare Life Sciences, Marlborough, MA) and PBS up to 2 ml followed by overnight on sepharose digestion at RT with gentle rotation. The cleaved untagged SHP-2 proteins were collected by 3 × 1 ml PBS washes, concentrated by Amicon Ultra-4, 10 K centrifugal filters and subjected to FPLC purification. The protein was subsequently purified as previously described^38^. Protein concentration was measured by a standard Bradford assay (Bio-Rad Laboratories).

### Assessment of SHP-2 interaction with PD-1 ITIM-pY223 and PD-1 ITSM-pY248 by electrophoretic mobility shift with Native PAGE

For these studies, SHP-2 amino acids 1–225, which only contains the tandem SHP-2 N-SH2 and C-SH2 domains in their natural tandem sequence (referred to as SHP-2 t-SH2) was generated. Binding with monophosphotyrosyl ITIM-pY223 (pITIM) or monophosphotyrosyl ITSM-pY248 (pITSM) peptide was assessed. Two micromolar of SHP-2 t-SH2 were mixed with peptides at various molar ratios in 50 mM HEPES, 100 mM NaCl, pH 7.4, in a final volume of 20 µl and incubated for one hour at room temperature. Five microliters 5x Native Sample Buffer were added and Native PAGE was performed at 4 ^o^C with 8–16% gradient Tris-Glycine gel (Bio-Rad) followed by Coomassie stain. Binding of t-SH2 with a phosphopeptide corresponding to the native PD-1 cytoplasmic tail containing phosphorylation of either both Y223 and Y248 (PD-1cyto-pITIM-pITSM), Y248 (PD-1cyto-ITIM-pITSM) or phosphorylation of Y223 (PD-1cyto-pITIM-ITSM) was assessed using the same approach. Peptide sequences are described in the section “Phosphopeptides” below.

### Surface plasmon resonance

Measurements were made using a Biacore 3000 (GE Healthcare) at the Dana-Farber Cancer Institute proteomics core facility. Phosphotyrosyl peptides pITIM (KEDPSAVPVFSVD(pY)GELDFQWRE) and pITSM (KTPEPPVPCVPEQTE(pY)ATIVFPS) were used. For immobilization, presentation of the peptide at high concentration was used to overcome the difficulties in immobilizing the negatively charged phosphopeptides (pI ≤ 3) to the negatively charged sensor chip^64^. In all, 2 mg/ml of phosphopeptide in 50 mM HEPES, 1 M NaCl, pH 7.5, was presented to the sensor chip surface, previously activated with N-hydroxysuccinimide (NHS) and 1-ethyl-3-(3-dimethylaminopropyl)-carbodiimide (EDC), for 7 min, followed by inactivation of the surfaces with ethanolamine. Removal of non-covalently bound peptides was achieved using a 3-min pulse of 2 M guanidine hydrochloride followed by re-equilibration in running buffer HBS-EP (GE Healthcare Life Sciences)^65^. Efficient immobilization of the phosphopeptides was confirmed by binding of phosphotyrosine-specific 4G10 monoclonal antibody (Upstate Biotechnology, Inc. Cat. Number 05-321). In our experiments, the average value for PD-1 phosphopeptide immobilization was 365 ± 82.4 RU corresponding to an estimated immobilized ligand concentration of 1.2 mM within the carboxydextran matrix (Conc_ligand_ = Response_ligand_/100 x  Mr_ligand_ (mol/liter)^66^ a value 3750x higher than the maximum SHP-2 concentration tested (320 nM). Negative control surfaces were prepared by performing the immobilization under identical conditions as above in the absence of both peptide and protein and used to correct for bulk refractive index signals introduced by the protein storage buffers. Experimental binding measurements were performed using HBS-P running buffer supplemented with 6.6 mM phosphate, to maintain identical PO_4_^3−^ concentration across all protein concentrations, and further supplemented with 10 mM DTT and adjusted to pH 7.4. After each cycle, the chip was regenerated with a 1 min pulse of 3 M NaCl and 1 min pulse of 6 M guanidine hydrochloride, pH 7.0 as previously described^67^. Neither loss of peptide or tyrosine phosphorylation (as assessed by anti-phosphotyrosine binding) nor change in baseline RU was apparent during the course of an assay. Sensograms of SHP-2 -full-length at 0, 20, 40, 80, 160, 320 nM, and of the single SH2 domains, N-SH2 and C-SH2, at 0, 1, 3, and 10 μM were collected for 900 s (15 min) association and 900 s dissociation time. Data were analyzed by BIAeval software (GE Healthcare Life Sciences) in each instance either determining separately the *k*_d_ (dissociation rate constant) followed by the *k*_a_ (association rate constant) and calculation of *K*_D_ = *k*_d_*/k*_a_ or by plotting *R*_Eq_ against concentration within the BIAeval package and using the “Steady-State Affinity” general fitting model to obtain the *K*_A_.

### Isothermal titration calorimetry

Isothermal titration calorimetry (ITC) was performed on a MicroCal ITC200 instrument (Malvern, Worcestershire, UK), using established methodology. Briefly, PD-1 phosphopeptides (PD-1cyto-pITIM-pITSM, PD-1cyto-ITIM-pITSM and PD-1cyto-pITIM-ITSM) and SHP-2 tandem-SH2 protein (t-SHP-2) were prepared in buffer containing 50 mM HEPES pH 7.4, 100 mM NaCl, 5 mM TCEP. Each peptide (50–100 µM) was titrated into a t-SHP-2 sample (3–5 µM) in a total of 19 injections at 20^ o^C. We used reference power 6.0, initial delay 60 s and stirring speed 750 rpm. Injection volume was 0.4 µl for first injection and 2 µl for subsequent injections, injection duration was 0.8 s for first injection and 4 s for subsequent injections, with 150 s spacing and 5 s filter period. Reference cell was filled with sterile Milli-Q water. Controls of peptide titrations into buffer were run for baseline corrections. Data analysis was done using Origin 7.0 software.

### Measurement of SHP-2 activity

Catalytic activity of SHP-2 was monitored by a fluorescent assay using the substrate 6,8-difluoro-4-methylumbelliferone (DiFMUP) as previously with slight modifications^38,68^. Specifically, the phosphatase reactions were performed in 50 mM HEPES, pH 7.4, 100 mM NaCl and 10 mM DTT in 96-well black polystyrene plate, flat bottom, non-binding surface, using a final reaction volume of 100 µl. In all, 1.6 µg/ml of SHP-2-WT or mutant proteins were incubated with 20 µM DiFMUP with the indicated concentrations of phosphotyrosyl peptides or without peptide at 37 ^o^C. For some experiments with highly SHP-2-activating peptides we used 0.5 µg/ml of SHP-2-WT so that activity measurements could fall within range. Fluorescence signal was monitored every 1 min for 3 min by a SpectraMax5 microplate reader and reaction rate was calculated by the change of fluorescence signal with time using excitation and emission wavelengths of 340 nm and 450 nm, respectively. Under these conditions, product formation was linear with respect to the time of incubation. Results were normalized against the basal activity determined for each condition in the absence of peptide and activity was calculated.

### Phosphopeptides

All peptides were synthesized at the Tufts University Core Facility. All PD-1 phosphopeptides corresponded to human PD-1 sequences. The sequence of the phosphopeptides used were as follows, with pY indicating the phosphorylated tyrosine.

Monophosphoryl ITSM: KTPEPPVPCVPEQTE(pY)ATIVFPS

Monophosphoryl ITIM: KEDPSAVPVFSVD(pY)GELDFQWRE

Bisphosphoryl ITSM peptide pITSM-Ahx4-pITSM (bpITSM): TE(pY)ATIVFP-Ahx4-QTE(pY)ATIVFPS

Bisphosphoryl ITIM peptide pITIM-Ahx4-pITIM (bpITIM): VD(pY)GELDFQ-Ahx4-SVD(pY)GELDFQW

Bisphosphoryl ITIM-ITSM peptide pITIM-Ahx4-pITSM (pITIM-pITSM): VD(pY)GELDFQ-Ahx4- QTE(pY)ATIVFPS

Bisphosphoryl ITSM peptide pITSM-Ahx1-pITSM (bpITSM-Ahx1): TE(pY)ATIVFP-Ahx1-QTE(pY)ATIVFPS

Bisphosphoryl ITSM peptide pITSM-Ahx2-pITSM (bpITSM-Ahx2): TE(pY)ATIVFP-Ahx2-QTE(pY)ATIVFPS

Bisphosphoryl ITSM peptide pITSM-Ahx10-pITSM (bpITSM-Ahx10): TE(pY)ATIVFP-Ahx10-QTE(pY)ATIVFPS

Bisphosphoryl peptide of native sequence of PD-1 cytoplasmic tail (PD-1cyto-pITIM-pITSM): SAVPVFSVD(pY)GELDFQWREKTPEPPVPCVPEQTE(pY)ATIVFPS

Monophosphoryl peptide of native sequence of PD-1 cytoplasmic tail (PD-1cyto-ITIM-pITSM): SAVPVFSVDYGELDFQWREKTPEPPVPCVPEQTE(pY)ATIVFPS

Monophosphoryl peptide of native sequence of PD-1 cytoplasmic tail (PD-1cyto-pITIM-ITSM): SAVPVFSVD(pY)GELDFQWREKTPEPPVPCVPEQTEYATIVFPS

Monophosphoryl IRS-Y727: TGD(pY)MNMSPVG

Monophosphoryl IRS-Y1172: SLN(pY)IDLDLVK

Bisphosphoryl IRS peptide 1172-Ahx4–1222 (bpIRS): LN(pY)IDLDLV-Ahx4-LST(pY)ASINFQK

### Cytokine production

Culture supernatants were harvested at the indicated time points and IL-2 concentration was assessed by ELISA (Biolegend or R&D Systems).

### Statistics and reproducibility

Statistical analysis was performed by Student’s *t*-test or ANOVA and Tukey’s multiple comparisons test. A *P*-value of <0.05 was considered statistically significant. Number of replicates per experiment and statistical values are indicated in the figure legends.

### Reporting summary

Further information on research design is available in the Nature Research Reporting Summary linked to this article.

## Supplementary information

Supplementary Information

Reporting Summary

Description of additional supplementary items

Supplementary Data 1

Peer Review File

## Data Availability

All data generated or analyzed during this study are included in the published article and its supplementary information files. Source data behind graphs are available as Supplementary Data 1.
