# Supplementary material for: Interaction of SHP-2 SH2 domains with PD-1 ITSM induces PD-1 dimerization and SHP-2 activation
Source: Commun Biol. 2020 Mar 17;3:128. doi: 10.1038/s42003-020-0845-0 (PMC7078208; doi:10.1038/s42003-020-0845-0)
Supplement: Supplementary file 1 — Supplementary Information [file 42003_2020_845_MOESM1_ESM.pdf]

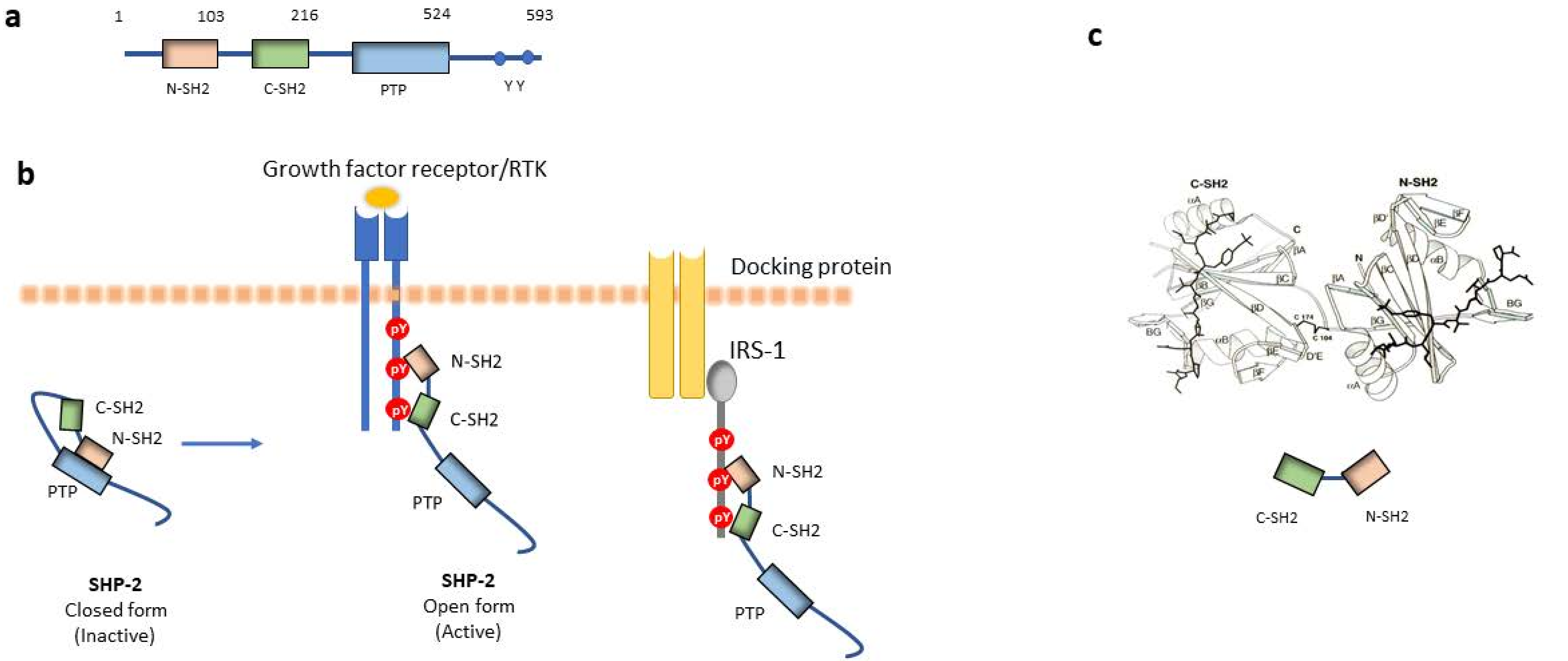

Supplementary Figure 1. Regulation of SHP-2 activation and unique structural properties of SHP-2 SH2 domains. (a) SHP-2 contains two tandem SH2 domains, N-terminal (N-SH2) and C-terminal SH2 (C-SH2), followed by a single phosphatase (PTP) domain, and a C-terminal hydrophobic tail with two tyrosine phosphorylation sites. **(b)** Exposure of cells to a variety of extracellular stimuli triggers the binding of SHP-2 via its SH2 domains to tyrosine-phosphorylated receptors for growth factors as well as to tyrosine-phosphorylated docking proteins such as insulin receptor substrates (IRSs). At the basal state, the N-SH2 domain of SHP-2 binds the phosphatase domain in an auto-inhibitory closed conformation and directly blocks its active phosphatase site. Interaction of the N-SH2 domain with phosphotyrosine peptide disrupts its interaction of N-SH2 with the phosphatase active site and activates the enzyme. The C-SH2 domain contributes binding energy and specificity but does not have a direct role in enzymatic activation. **(c)** The two SH2 domains of SHP-2 have a relatively fixed and roughly antiparallel orientation relative to one another, with the phosphopeptide-binding sites lying on the surface of the molecule and widely spaced. This relative fixed orientation of SH2 domains is stabilized by a disulphide bond and a small hydrophobic patch within the interphase that separates the phosphopeptide binding sites.

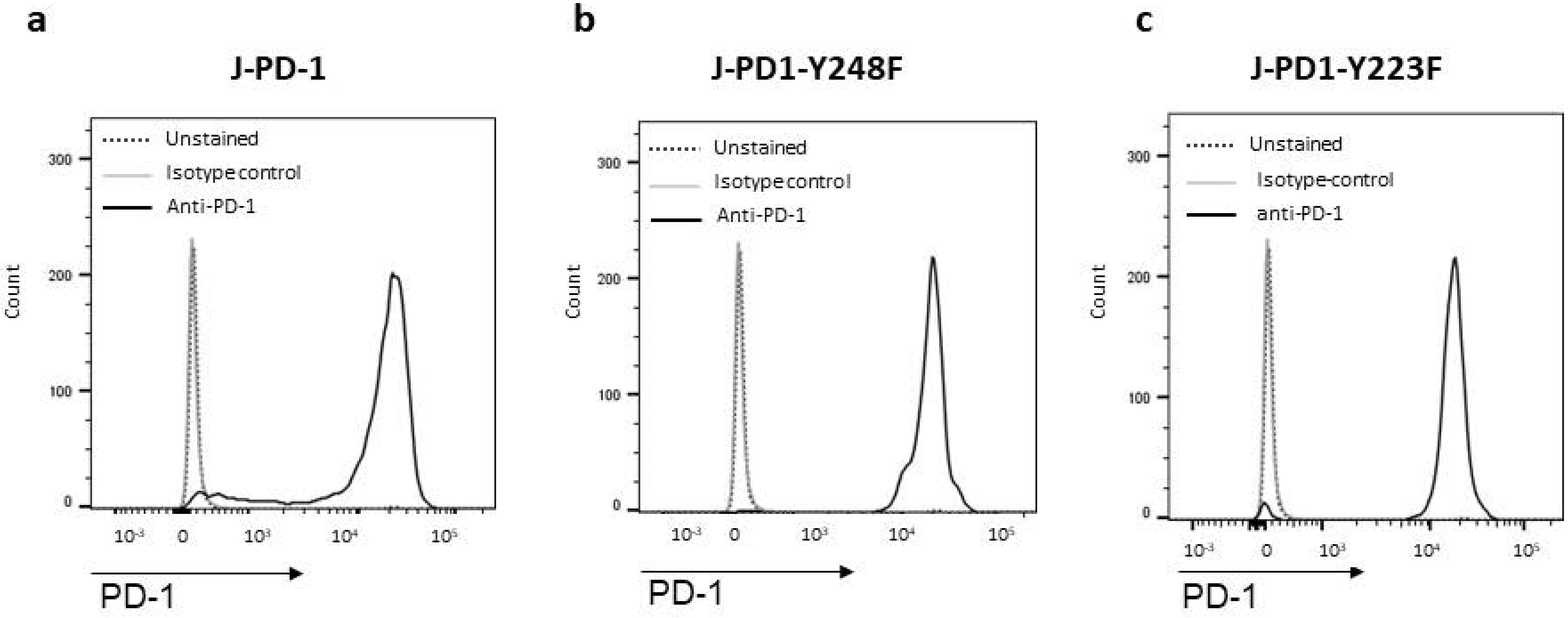

Supplementary Figure 2. Generation of PD-1-expressing J-PD-1, J-PD-1Y248F and J-PD1Y223F cells. (a-c) Jurkat T cells were transfected with human PD-1 cDNA or human PD-1 cDNA in which tyrosine 248 or tyrosine 223 was mutated to phenylalanine expressed in pEF6 vector and stable PD-1$^+$ cell lines and clones were generated by antibiotic selection. Experiments were performed in polyclonal cell lines and clones. Surface expression of PD-1 in subclones J-PD-1, J-PD1-Y248 and J-PD1-Y223 is shown.

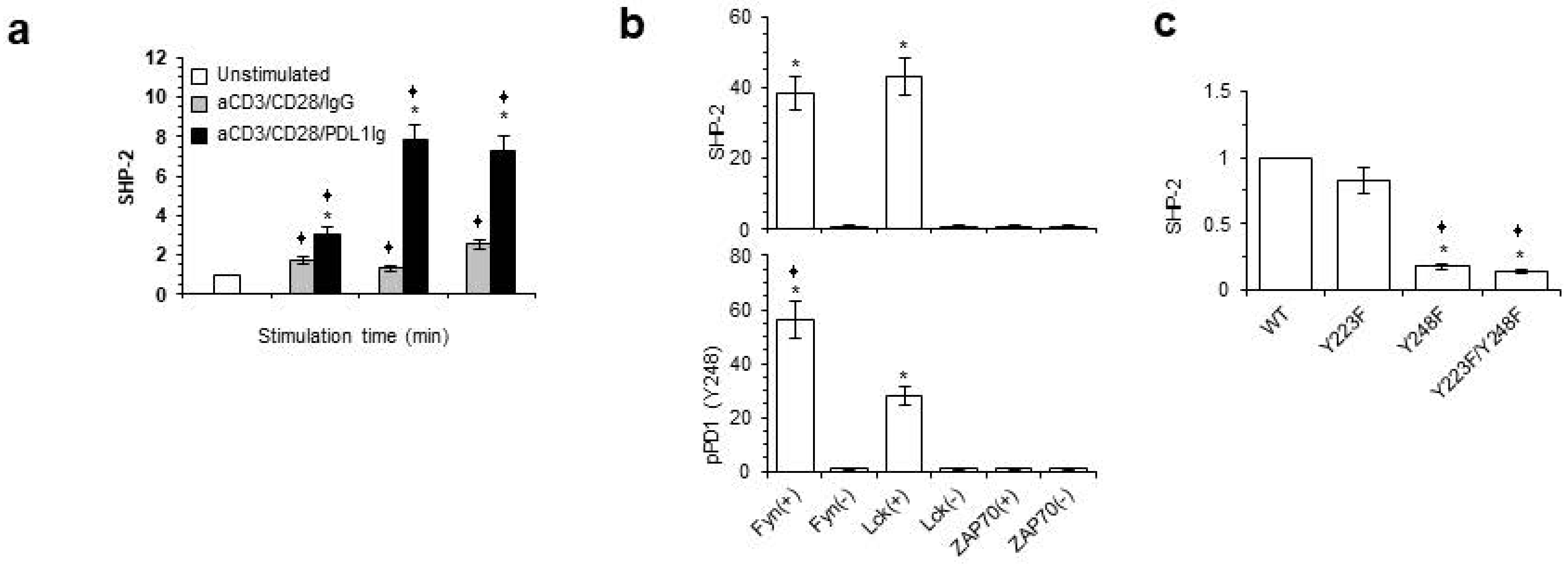

Supplementary Figure 3. **(a) Densitometric analysis of Figure 1a.** The abundance of SHP-2 normalized to that of immunoprecipitated PD-1 at each time point and expressed as the fold change over the levels obtained in unstimulated cells (defined as 1). Fold changes are compared between unstimulated and stimulated cells at each time point (♦P < 0.05) or between cells stimulated with or without PD-1 ligation at each time point (*P < 0.05). Data are presented as the means ± SEM, n = 5 experiments. **(b) Densitometric analysis of Figure 1b**. In cells transfected with kinase active (+) or inactive (-) Fyn, Lck or ZAP70, the abundance of SHP-2 and pPD1(Y248) normalized to that of immunoprecipitated PD-1 was expressed as the fold change over the values obtained with the kinase inactive (-) form (defined as 1). Fold changes were compared between the corresponding kinase active and inactive forms (*P < 0.05) or between the Fyn active and Lck active forms (♦P < 0.05). Data are presented as the means ± SEM, n = 5 experiments. **(c) Densitometric analysis of Figure 1c**. The abundance of SHP-2 normalized to that of immunoprecipitated PD-1 in each condition was expressed as the fold change over the values obtained in samples expressing PD-1-WT (defined as 1). Fold changes are compared to the values obtained in samples expressing PD-1-WT (*P < 0.05) and in samples expressing PD-1-Y223F (♦P < 0.05). Data are presented as the means ± SEM, n = 5 experiments.

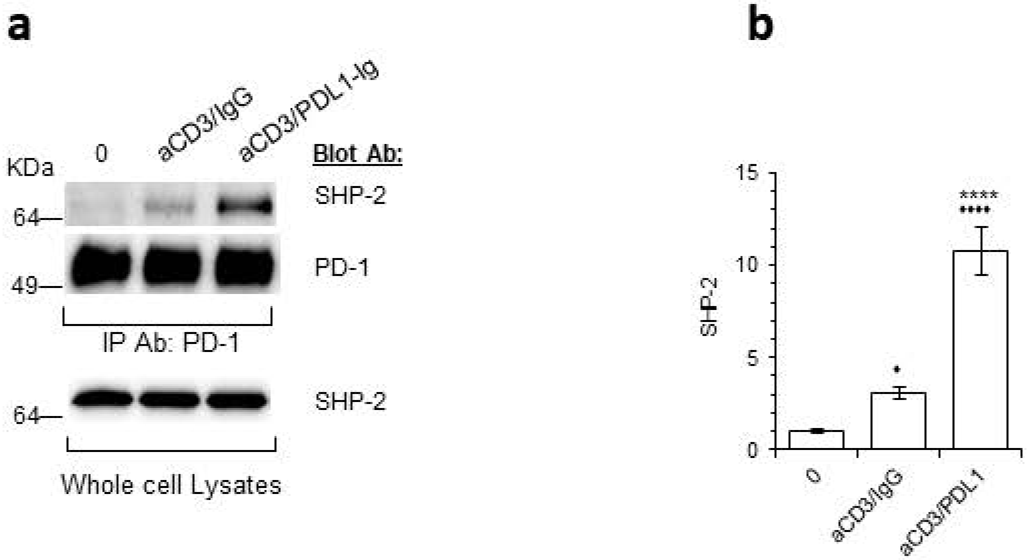

Supplementary Figure 4. TCR/CD3 signaling together with PD-1 ligation is required for PD-1 interaction with SHP-2 (a) Jurkat-PD1 cells were left unstimulated (0) or stimulated with beads coated with aCD3/IgG or aCD3/PDL1-Ig for 5 min. Lysates were prepared followed by immunoprecipitation with anti-PD-1 antibody, SDS-PAGE and western blot with antibodies for SHP-2 and PD-1. SHP-2 expression in whole cell lysates was also examined. **(b)** Densitometric analysis of Figure S4a. The abundance of SHP-2 normalized to that of immunoprecipitated PD-1 in each condition and expressed as the fold change over the levels obtained in unstimulated cells (defined as 1). Fold changes are compared between unstimulated and stimulated cells at each condition (⁺P < 0.05), (⁺⁺⁺⁺P < 0.0001) or between cells stimulated with or without PD-1 ligation (****P < 0.0001). Data are presented as the means ± SEM, n = 5 experiments

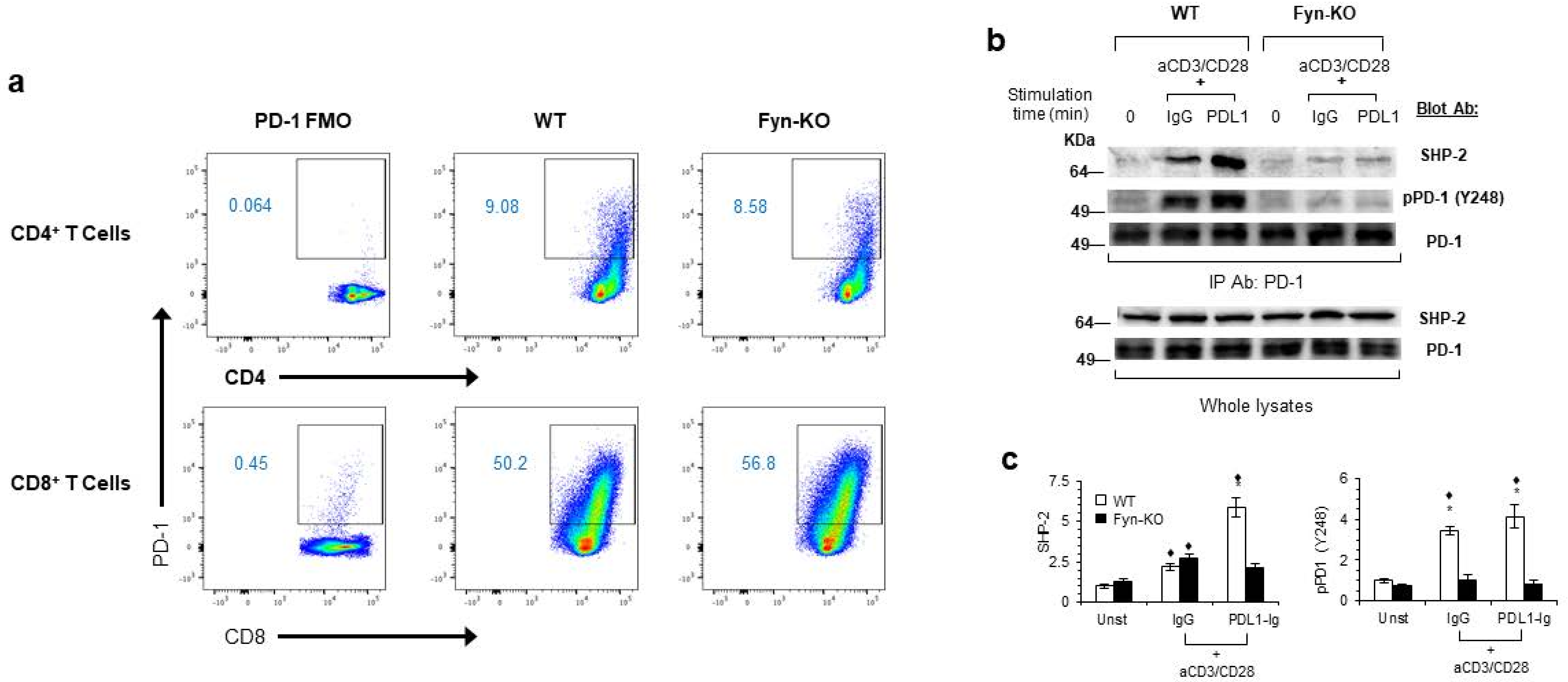

Supplementary Figure 5. Fyn is required for PD-1(Y248) phosphorylation and interaction with SHP-2. (a) T cells purified from spleens and lymph nodes of WT and Fyn-KO mice were cultured for 72h with anti-CD3 and anti-CD28 mAb and expression of PD-1 was examined by flow cytometry. **(b)** After resting in RPMI and 2% FBS, the cells were either left unstimulated (0) or stimulated with aCD3/CD28/IgG- or aCD3/CD28/PDL1-Ig-coated beads for 3 minutes. Cell lysates were prepared followed by immunoprecipitation with anti-PD-1 antibody, SDS-PAGE and western blot with antibodies specific for SHP-2, pPD-1(Y248) and PD-1. Expression of PD-1 and SHP-2 in whole cell lysates was also examined. **(c)** Densitometric analysis of the immunoprecipitation data shown in **(b)**. The abundance of SHP-2 and pPD1(Y248) was normalized to that of immunoprecipitated PD-1 and was expressed as fold change over the values obtained in unstimulated (0) WT cells (defined as 1). Fold changes are compared between unstimulated and stimulated cells in each strain (♦$P < 0.05$) and between WT and Fyn-KO cells in each stimulation condition (*$P < 0.05$). Data are presented as the means ± SEM, n = 3 experiments.

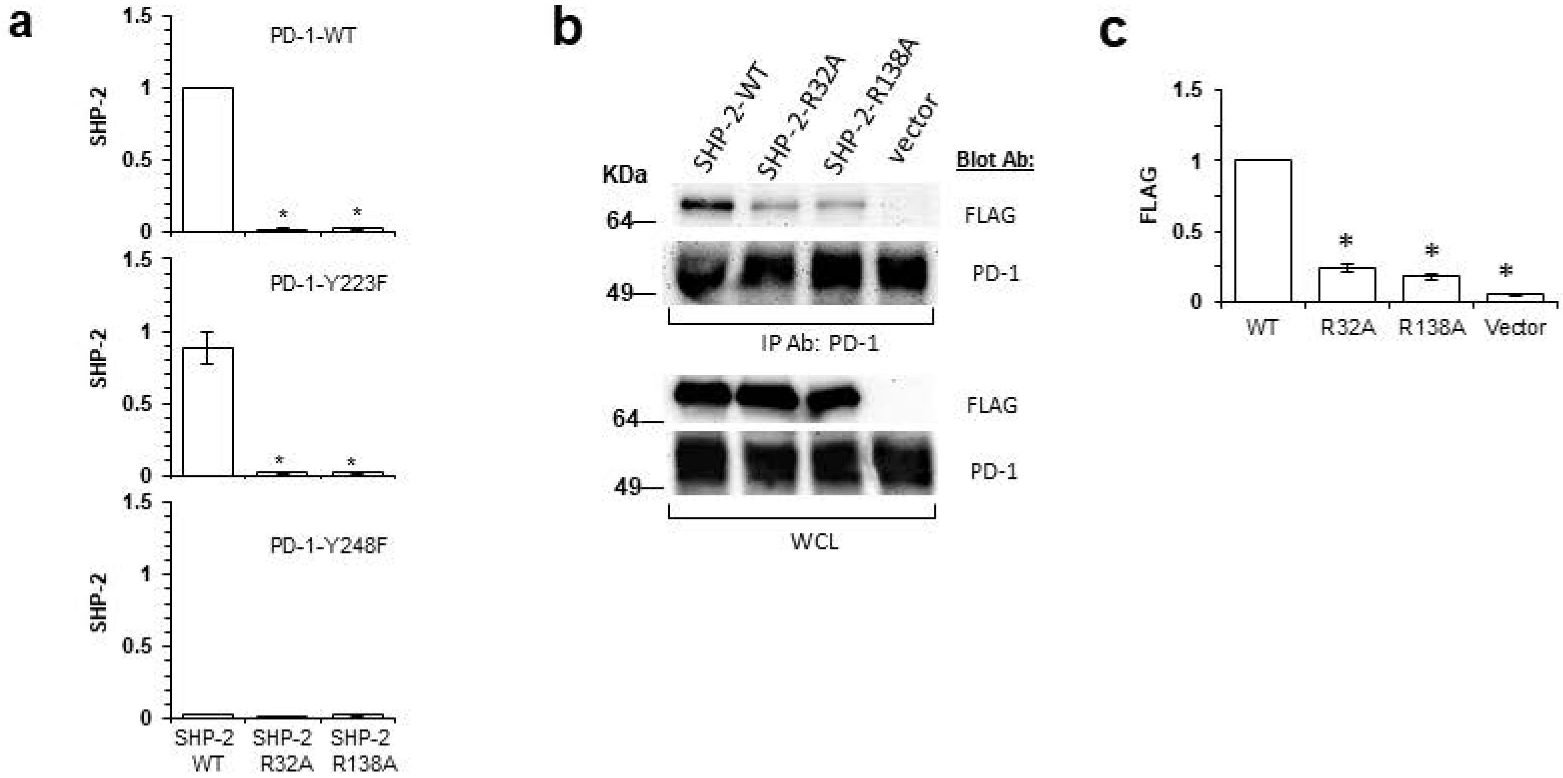

Supplementary Figure 6. Both SH2 domains of SHP-2 can mediate SHP-2 binding to PD-1. (a) Densitometric analysis of Figure 2a. COS cells were transfected with kinase active Fyn, PD-1 WT and either SHP-2-WT, SHP-2-R32A or SHP-2-R138A mutants FLAG-tagged; Fyn, PD-1-Y223F and either SHP-2-WT, SHP-2-R32A or SHP-2-R138A; Fyn, PD-1-Y248F and either SHP-2-WT, SHP-2-R32A or SHP-2-R138A. Immunoprecipitation of cell lysates was performed with anti-PD-1 antibody followed by SDS-PAGE and immunoblot with FLAG- or PD-1-specific antibodies. In each sample, the abundance of SHP-2 WT (FLAG) coprecipitated with PD-1 was normalized to that of immunoprecipitated PD-1 and was expressed as fold change over the value obtained in cells transfected with SHP-2 WT and PD-1 WT (defined as 1). Fold changes of SHP-2 immunoprecipitated with PD-1 were compared among cells transfected with SHP-2 WT, SHP-2 R32A or SHP-2 R138A (*$P < 0.05$). Data are presented as the means ± SEM, n = 4 experiments. **(b)** Primary human T cells were activated for 72h with aCD3 (100 ng/ml) and aCD28 (300 ng/ml) mAbs. The cells were then transfected with the indicated FLAG-tagged SHP-2 constructs or empty vector as described in Methods, cell lysates were prepared and PD-1 immunoprecipitation was performed followed by SDS-PAGE and immunoblot with FLAG- or PD-1-specific antibody. **(c)** Densitometric analysis of figure (b). The abundance of SHP-2 (FLAG) was normalized to that of immunoprecipitated PD-1 in each condition and was expressed as the fold change over the values obtained in samples transfected with SHP-2-WT (defined as 1). Fold changes are compared to the values obtained in samples transfected with SHP-2 WT (*$P < 0.05$, n = 3 experiments). WCL: whole cell lysates.

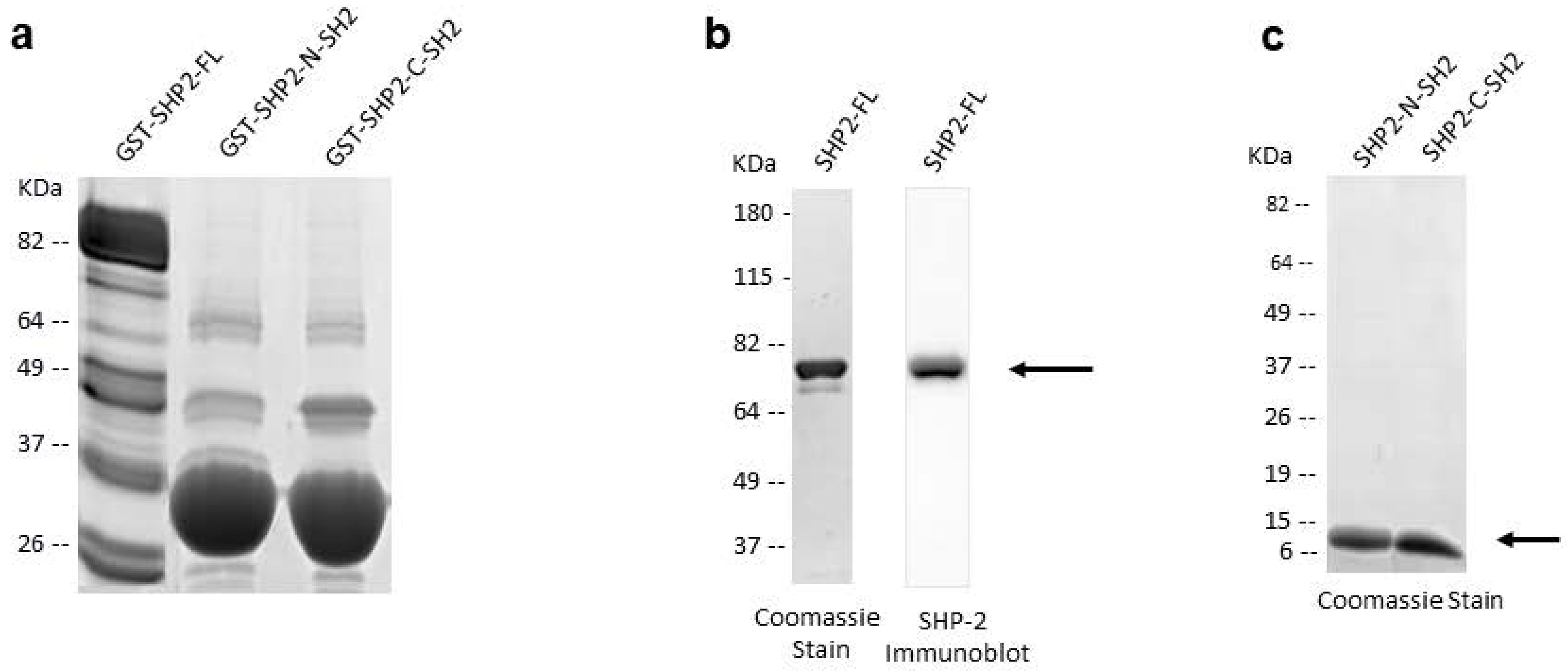

Supplementary Figure 7. Production and purification of SHP-2-FL, SHP-2-N-SH2 and SHP-2-C-SH2 proteins. (a) The GST fusion proteins GST-SHP-2-WT, GST-SHP-2-N-SH2 and GST-SHP-2-C-SH2 were generated, purified by affinity chromatography using glutathione-Sepharose beads and analyzed by SDS-PAGE and Coomassie staining. **(b-c)**. The GST tag was removed by thrombin digestion. The cleaved untagged proteins were collected, concentrated, subjected to FPLC purification and subsequently applied to anion exchange column. The eluted fractions were subjected to SDS-PAGE and Coomassie staining. SHP-2 FL was also assessed by immunoblotting (b), whereas the truncated SH2 domains (c) are not recognized by SHP-2-specific antibodies and cannot be detected by immunoblot.

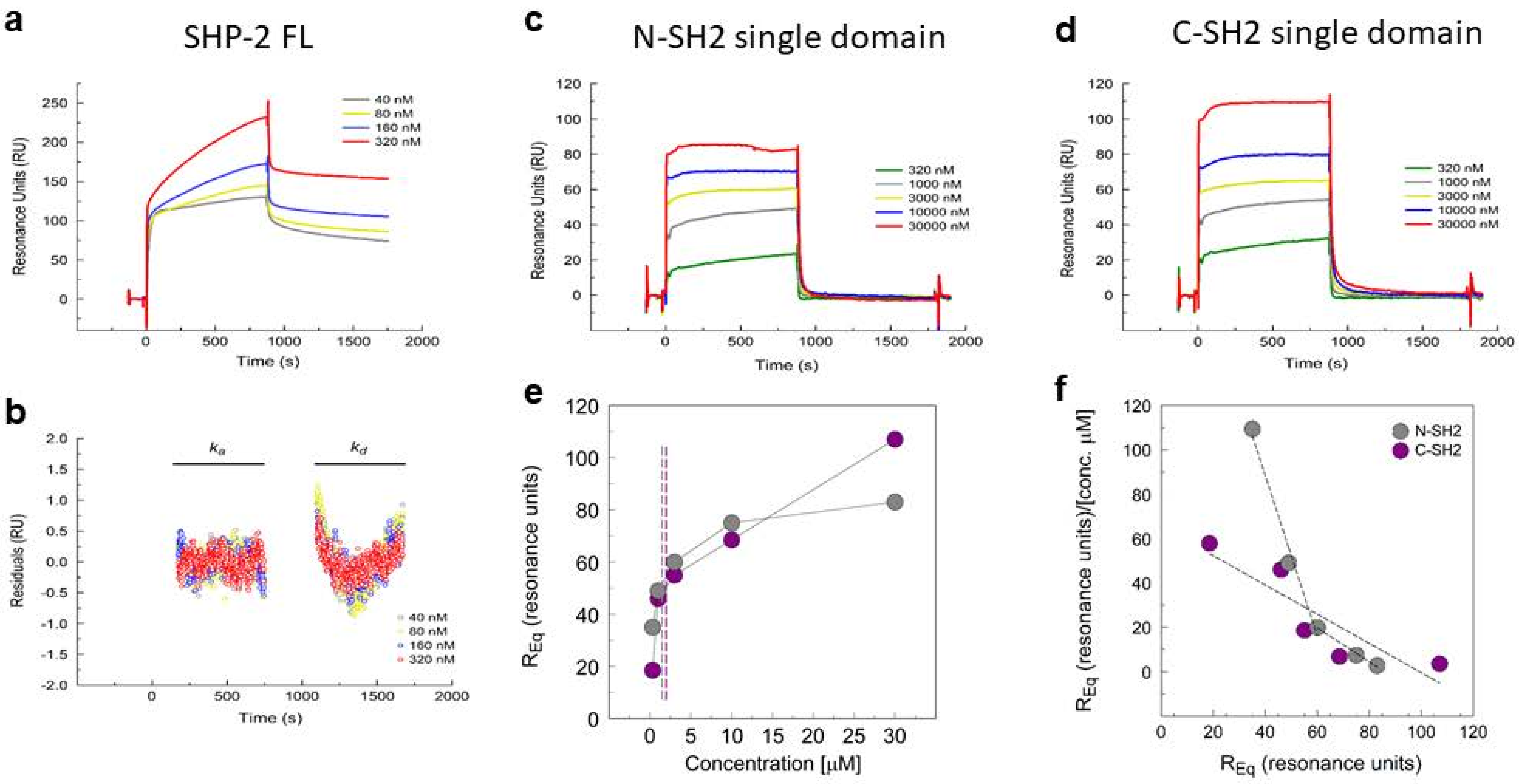

Supplementary Figure 8. Dose-response curves for SHP-2-full length (FL), N-SH2 domain, and C-SH2 domain binding to ITSM-pY248 determined by SPR, and derivation of kinetic parameters. All SHP-2-related analytes were passed over the pITSM-p248 phosphopeptide ligand-immobilized surface using standard conditions as described in the Methods section. **a.** For SHP-2 FL, the dissociation constant (k_d) was first determined for each analyte concentration using the BIAeval package and was then used to discriminate the association constant k_a during the association phase. Calculated K_D was then determined as k_d/k_a. The Chi-square value, where smaller values equate to better fits, were 0.06 for k_a and 0.06 for k_d. **b.** Following the curve fitting (in **a**), the residuals ([Predicted – Observed] RU) were calculated with a uniform variation of 0.5 – 1 RU range for variation from the fitted curves (colors correspond to those used in **(a)**. **c, d.** Binding curves for SHP-2 N-SH2 and C-SH2 domains to ITSM-pY248 (representative of 4 experiments). **e.** R_{Eq}/dose response curve for single domain binding; dashed lines indicate calculated steady state K_D for N-SH2 (grey) and C-SH2 (purple) domains (from **c, d**). **f.** Scatchard reciprocal transformation analysis of curves in **(e)** where, assuming a linear model and fit, slope = -1/K_D. Note that N-SH2 domain binding clearly represents a “concave-upward” curve indicating lack of fit to a 1:1 binding model.

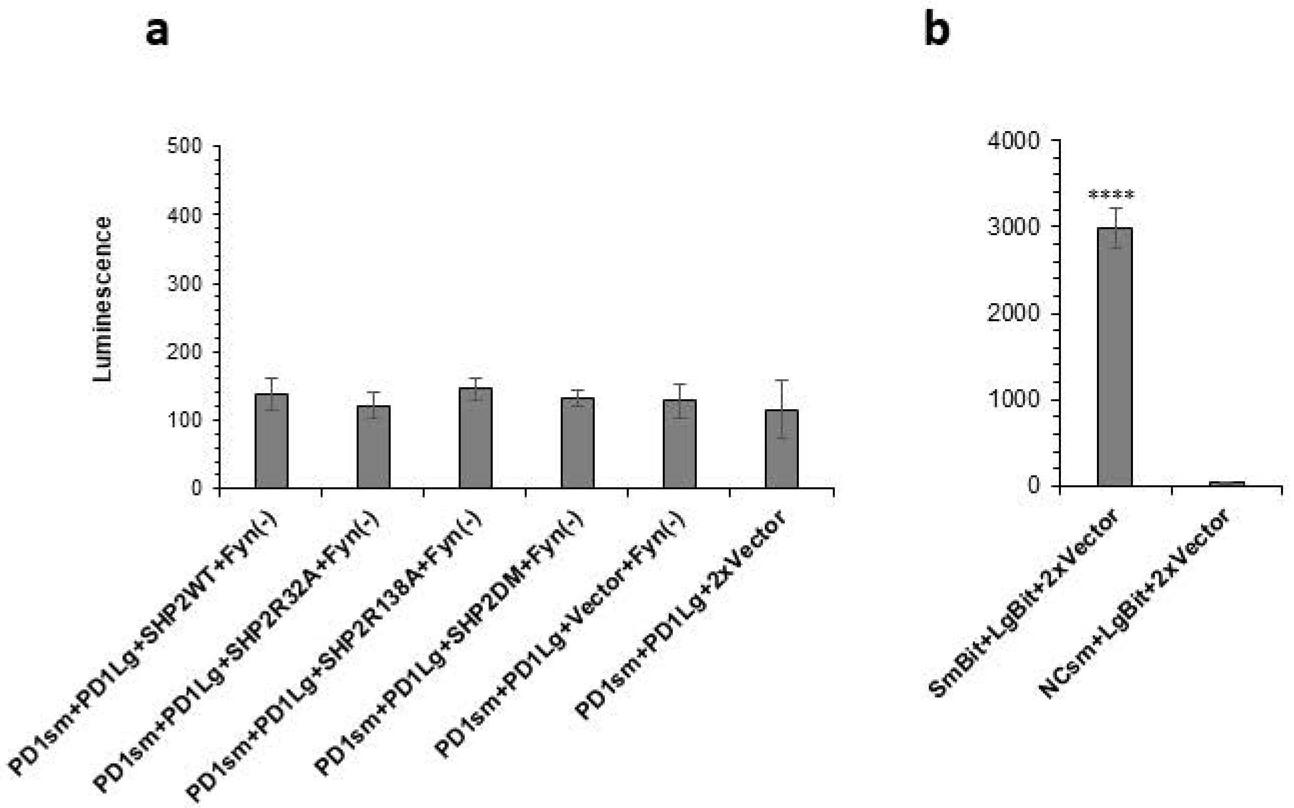

Supplementary Figure 9. SHP-2 mediates bridging of two PD-1 molecules in the presence of kinase active but not kinase inactive Fyn. (a) HEK-293 cells were co-transfected with PD-1-SmBiT and PD-1-LgBiT together with kinase inactive (-) Fyn and either SHP-2 WT, SHP-2-R32A, SHP-2-R138A or double mutant SHP-2-R32A/R138A (DM), or control empty vector. Complex formation between PD-1-SmBiT and PD-1-LgBiT was assessed by luciferase assay. (**b)** As positive and negative control for the assay, cells were transfected with SmBit+LgBit or NCsm+LgBit (provided by the NanoBit Assay system), respectively, together with empty vector for normalization of cDNA expression to experimental samples. Comparison of complex formation among the different conditions was performed by 2way ANOVA and Tukey's multiple comparisons test for panel (a) or Student's T-test for panel (b) and significant differences are indicated (****$p<0.0001$, Student's T-test, n=6 experiments for all results shown).

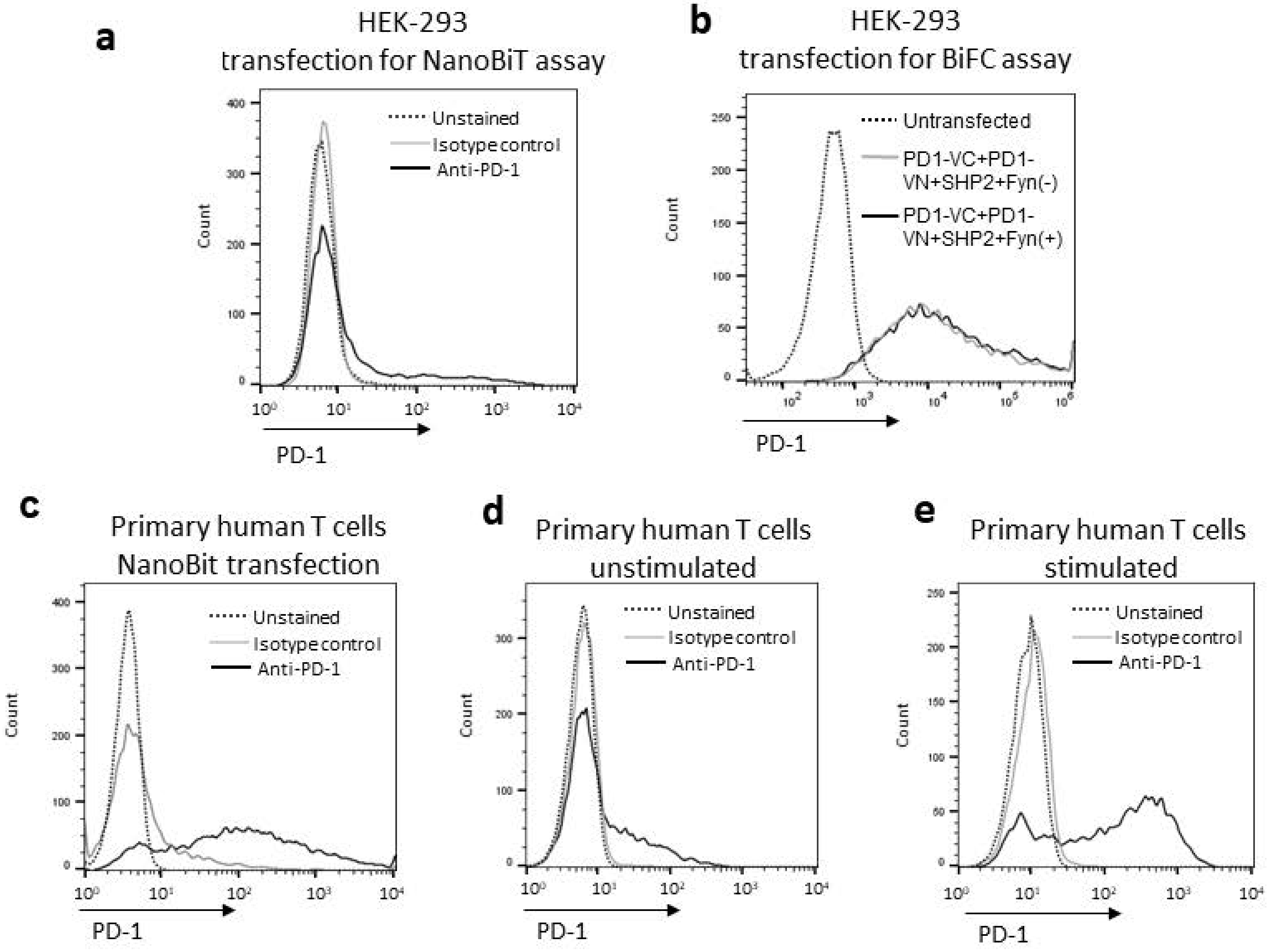

Supplementary Figure 10. (a) HEK-293 cells were transfected with PD1Lg and PD1sm NanoBit constructs and expression of PD-1 was assessed by flow cytometry. **(b)** HEK-293 cells were transfected with PD1-VC, PD1-VN, SHP-2 and either Fyn active (+) or inactive (-) kinase and expression of PD-1 was assessed by flow cytometry. **(c)** Primary human T cells were transfected with PD1Lg and PD1sm NanoBit constructs and expression of PD-1 was assessed by flow cytometry. **(d)** Expression of PD-1 in primary human T cells before stimulation. **(e)** Expression of PD-1 in primary human T cells after stimulation for 48 hours with anti-CD3 and anti-CD28 antibodies. Results are representative of three independent experiments.

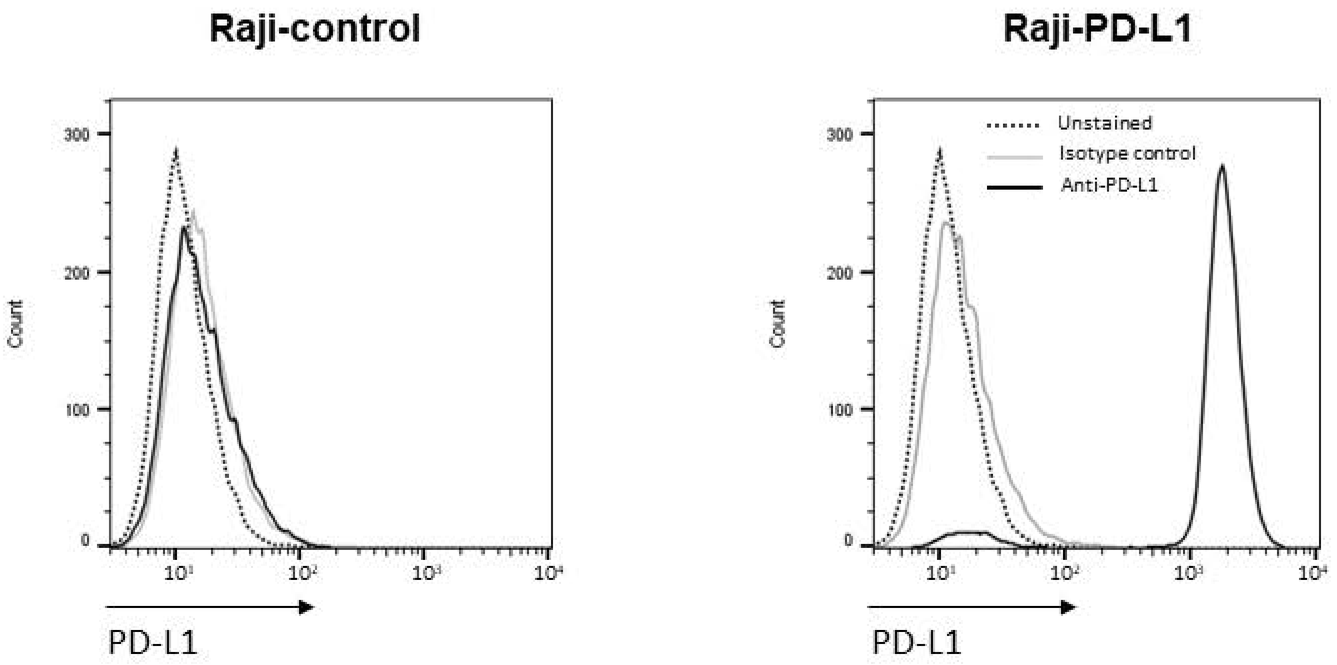

Supplementary Figure 11. Generation of Raji-PD-L1. Raji cells were transfected with either empty vector (control) or human PD-L1 cDNA and stable lines were generated by antibiotic selection. Cell lines were subcloned and stable clones were generated. Expression of PD-L1 was examined by flow cytometry.

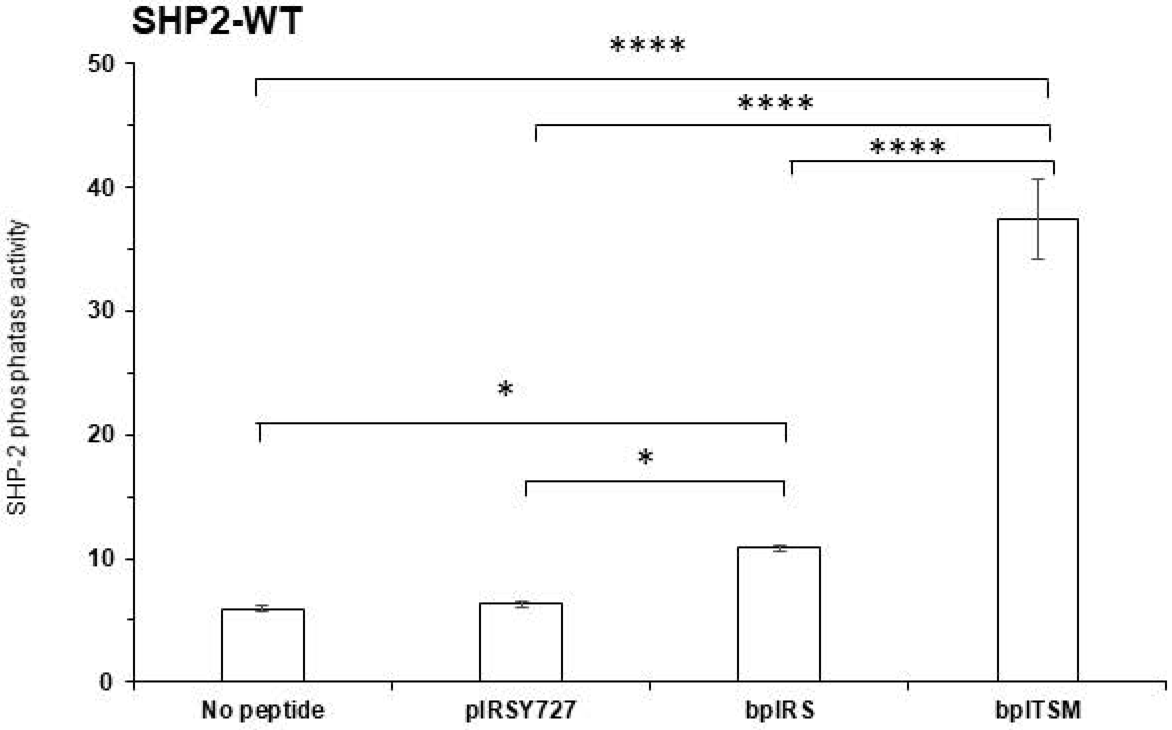

Supplementary Figure 12. Bisphosphorylated PD-1 ITSM pY248 induces higher SHP-2 enzymatic activity compared to dimeric bisphosphorylated IRS1. 1.6 µg/ml of purified SHP-2-WT protein was incubated with 20 µM DiFMUP with or without the indicated phosphotyrosyl peptides at a concentration of 20 nM. Monophosphoryl peptide pIRSY727 was used as negative control. SHP-2 phosphatase activity was monitored by a fluorescent assay using 6,8-difluoro-4-methylumbelliferone (DiFMUP) as substrate. Fluorescence signal was monitored at 37°C every 1 min for 3 min and reaction rate was calculated by the change of fluorescence signal with time by a microplate reader using excitation and emission wavelengths of 340 nm and 450 nm, respectively. Comparison of SHP-2 phosphatase activity among the different conditions was performed by 2way ANOVA and Tukey's multiple comparisons test and significant differences are indicated (*p<0.05, n=3 experiments, ****P<0.0001, n=3 experiments).

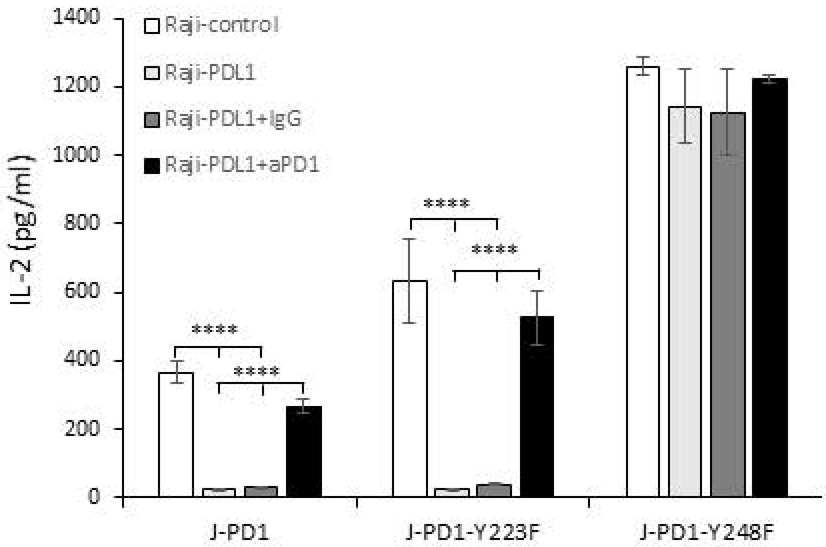

Supplementary Figure 13. Jurkat T cells stably expressing PD-1-WT (J-PD1), PD-1-Y223F (J-PD1-Y223F) or PD-1-Y248F (J-PD1-Y248F) were co-cultured with Raji-control or Raji-PD-L1 cells loaded with SEE. Where indicated, anti-PD-1 blocking antibody or isotype control was added in the cultures. Culture supernatants were collected at 24 hours and IL-2 production was measured by ELISA (****P<0.0001, n=3 experiments).
