# Supplementary material for: Interaction of SHP-2 SH2 domains with PD-1 ITSM induces PD-1 dimerization and SHP-2 activation
Source: Commun Biol. 2020 Mar 17;3:128. doi: 10.1038/s42003-020-0845-0 (PMC7078208; doi:10.1038/s42003-020-0845-0)
Supplement: Supplementary file 3 — Description of additional supplementary items [file 42003_2020_845_MOESM3_ESM.pdf]

Description of additional supplementary items

Supplementary Data 1

This file contains all source data underlying the graphs and full gel scans.
