# Supplementary material for: Interaction of SHP-2 SH2 domains with PD-1 ITSM induces PD-1 dimerization and SHP-2 activation
Source: Commun Biol. 2020 Mar 17;3:128. doi: 10.1038/s42003-020-0845-0 (PMC7078208; doi:10.1038/s42003-020-0845-0)
Supplement: Supplementary file 5 — Peer Review File [file 42003_2020_845_MOESM5_ESM.pdf]

Reviewers' comments:

Reviewer #1 (Remarks to the Author):

Patsoukis et al Interaction of SHP-2 SH2 domains with PD-1 phospho-ITSM bridges two PD-1 molecules
and can induce SHP-2 activation

This is an interesting study which provides new insights the PD-1 inhibitory receptor, which in important therapeutic target that has attracted huge interest in recent years.

The authors use a range of cellular and biochemical approaches to support a model in which two phosphorylated PD-1 receptors simultaneously bind to and activate a sing SHP2 molecule.

Their findings help reconcile previously puzzling results and raise interesting questions regarding whether and how PD-1 is dimerized upon ligand engagement.

The study is well written with few if any errors, and the Figures are clear and convincing.

Some minor points:

Line 108/109 it states that the '..PD-1: SHP-2 interaction was induced by CD3 ligation with or without CD28 costimulation'. No experiments are shown where there is no anti-CD28 antibody included in the stimulation.

Line 142-145: The sentence "Importantly, PD-1 ITSM-Y248F disrupted the interaction of PD-1 with either SHP-2 mutant (Fig. 2a, bottom panel and Supplementary Fig. S5a, bottom panel), suggesting that binding of each SH2 domain of SHP-2 with PD-1 when co-expressed in live cells depends on PD-1 ITSM-Y248" seems illogical. Since, as noted in the previous sentence, neither SHP-2 mutant bound WT PD-1, surely their lack of binding to PD-1 ITSM-Y248F tells you nothing extra.

Lines 153-7: "Consistently, compared with a peptide containing both phosphorylated tyrosines of the native PD-1 cytoplasmic tail (Fig. 2c, top panel), electrophoretic mobility shift of t-SHP-2 was preserved by a peptide containing phosphorylation of Y248 (PD-1cyto-ITIM-pITSM) but not by a peptide containing phosphorylation of Y223 (PD-
cyto-pITIM-ITSM) (Fig. 2c middle and bottom panel)." In fact, looking at Fig. 2c, it seems that the pITIM, while not enough on its own for binding, does seem to enhance binding mediated by pITSM.

Lines 192-3: "The affinity of each individual SH2 domain is negligible compared with the calculated affinity of SHP-2-full length (N-SH2 KD=2.2 μM, C-SH-2 KD=161 μM, SHP-2 KD=0.0197 μM) (Supplementary Fig. S7a, b, c)."
These Kd values do not seem to match the raw data shown in Fig. 7b and c. These data clearly show that the C-SH2 single domain is binding to a comparable level than the N-SH2 single domain when injected at similar concentrations, so it cannot be correct that the affinity of the C-SH2 single domain is 75 fold lower. I suspect they have obtained unreliable results because they have tried to fit ka and kd values and calculated the Kd. This is unreliable with low affinity interactions with fast kinetics. They should use a plot of steady state binding versus concentration to determine the Kd for these interactions.

What is the relationship between Fig 5f and S12, which look very similar? The authors should clarify this and perhaps consider ommitting S12.

Reviewer #2 (Remarks to the Author):

The work by Patsoukis et al is focused on the early mechanisms of PD-1 signaling via SHP-2 recruitment and activation. The topic is of course of interest to a wide audience, is timely and has the potential to impact translational work.

PD-1 contains two tyrosine-based structural motifs, and ITIM and an ITSM. Several studies have now shown convincingly that PD-1 phosphorylation (pTyr) recruits SHP-2, a cytoplasmic phosphatase, which then dephosphorylates multiple other transmembrane receptors. Some reports also have suggested a role for a PD-1/SHP-1 interaction, but I largely agree with the authors that SHP-2 appears to be the key player, based on the published data.

Upon PD-1 activation, a yet unexplored mechanism leads to pTyr of its two Tyr-based signaling motifs, ITIM and ITSM, which results in SHP-2 recruitment. The work by Patsoukis et al revolves around a fundamental second step: SHP-2 activation. Once SHP-2 is recruited to PD-1, via interaction of its SH2 domains with PD-1 pTyr, SHP-2 catalytic phosphatase domain is released from an auto-inhibitory conformation resulting in activation of the phosphatase activity. The mechanism of SHP-2 activation has been extensively studied. SHP-2 contains two tandem SH2 domains, N-term and C-term, followed by a C-terminal protein tyrosine phosphatase (PTP) domain. An intramolecular interaction between N-SH2 and the PTP leads to reduced PTP activity. Binding of N-SH2 to a target pTyr has the potential to release the PTP from its intramolecular interaction and thereby enhance PTP activity. The authors very clearly explain all of this in the introduction.

The authors move on to investigate in more detail the interaction properties between PD-1 and SHP-2 and focus on two key aspects. First, the authors conclude that SHP-2 bridges two PD-1 molecules via the pTyr Y248 PD-1 residue (using a luminescence-based dimerization reporter system), in other words, SHP2 would dimerize PD-1 upon PD-1 pTyr. Secondly, the authors also provide more evidence for the SHP-2 interaction with PD-1 Y248 by using SPR and ITC. The measurements that support the second observation are robust but not novel, while the data that supports the dimerization of PD-1 upon SHP-2 recruitment is, I found, incomplete and inconclusive at this stage. The novelty of this work lies in this newly proposed mechanism of PD-1 SHP-2-induced dimerization, however the work feels slightly rushed and premature.

Main concerns:
- In Figure 1 and 2 (and accompanying supplementary figures) the authors nicely show that the SHP-2/PD-1 interaction is largely mediated by PD-1 ITSM-Y248F, in good agreement with previous reports. The authors move on to investigate the contribution of the N-SH2 and C-SH2 for the PD-1/SHP2 interaction by performing pull-downs with PD-1 and R32A or R138A SHP-2 mutants. From these experiments the authors conclude that both domains are required for SHP-2 to interact with PD-1. The authors thus propose a model where both SH2 domains interact with pY248, whereby the N-SH2 domain binds one PD-1 molecule and the C-SH2 domain binds another PD-1 molecule also via the pY248. The authors conclude that this hypothesis explains why the Y248F mutation on PD-1 eliminates interaction with either SHP-2 WT or both SHP-2 SH2 mutants. Importantly, in data shown in Supplementary Fig. S7b, c, and which I think should be in the corresponding main Figure 3, the

authors show that the affinity of the N-SH2 domain to pY248 is 75-fold higher than that of the C-SH2 domain. Notice that the affinity of the N-SH2 is (according to authors "negligible") 100-fold lower than the affinity of the SHP-2-full length. The experiment is however incomplete, a second set of measurements should have been made with doubly pTyr PD-1 peptide, pY223 and pY248. Is it possible that binding to pY223, once the stronger N-SH2 binding to pY248 is established, can further stabilize the PD-1/SHP-2 interaction? Notice that even the extremely weak C-SH2 interaction with pY248 (KD=161 μM) contributes with a 100-fold increase in affinity over N-term alone (N-SH2 KD=2.2 μM V WT SHP2 KD=0.0197 μM). It is possible that a weak pY223 can stabilize the binding to WT SHP-2, once one of the SH2 domains is bound to pY248. This is important because if that is the case then an intra-molecular interaction between the SH2s of SHP-2 and PD-1 is much more likely to occur than an interaction between the SH2s of SHP-2 and two PD-1 molecules diffusing independently and particularly when one of the interactions (that mediated by the C-term SH2) is so weak, > 150 μM. In other words, the authors clearly show that the interaction between SHP2 SH2 is mediated by Y248; Y223 interaction is negligible to non-existent on its own, but what about Y223 in the context of Y248? Intramolecular avidity effects can drastically increase weak affinities, and as such this possibility should be accounted for. Given that this experiment was made for the ITC and that it showed that one PD-1 molecule can bind to SHP-2 using both pTyr 223 and 248, it would be important to include the same measurement in the context of the SPR work as well. Otherwise the authors are providing two conflicting pieces of data and it may appear that one result is being favored over the other.

- In the intracellular, cytoplasmic, environment KCl concentration is higher than NaCl. Given the low affinities the authors are reporting here (> μM), would it be possible to consider repeating some of the measurements in a buffer similar to: 10mM Hepes, pH 7.4, 146 mM KCl, and 4 mM NaCl 1mM DTT ? John Kuriyan and others have shown that for largely electrostatic mediated interactions, such as those established between SH2/pY, the salt composition of the buffer can have an important effect.

- The NanoBiT proximity assay has a very important caveat which the authors do not seem to consider. How confident are the authors that the luminescence signal is from the cell membrane and not from, for example, the ER? A FRET reporter, whereby the source of the signal could be confirmed could be useful. Regardless of the reporter used it is important to confirm the main source of the signal, particularly because the proteins under study are being overexpressed. This is even more important when some of the data appears to be slightly puzzling. For example, dimeric recombinant PD-L1 binding alone does not seem to induce a significant increase in PD-1 dimerization. Expression of kinase active Fyn seems to be required for dimerization. But shouldn't PD-L1 dimer be enough? This raises questions in terms of how much signaling is derived from surface membrane PD-1 and/or what is the assay sensitivity? It is also concerning that the expression of the PD-1Lg and PD1SM nanobit appears very low (Fig. S9a), which may hint at problems in protein stability. WT PD-1 expresses to very high levels (see Fig S2 and S4), while the nanobit PD1 constructs show extremely low levels of expression, even in HEK-293s. Perhaps the authors could perform an intracellular staining of PD-1 in HEK and Primary cells to confirm the ratio of surface:intracellular PD-1-nanobit construct.

- Later on, the authors claim that SHP-2 activation requires binding of both SH2 domains to "release" the inhibitory state of the catalytic PTP site. This may not be entirely correct. Hayashi et al, Cell Reports, 2017, show that a single, monovalent, interaction between a pY peptide and the N-term SH2 in SHP-2 is necessary and sufficient to induce strong enzymatic activation of SHP-2 using a particular peptide (https://www.sciencedirect.com/science/article/pii/S2211124717312226?via=ihub). Have the authors tested the PTP activity in the presence of pITSM alone? This is not clear from the data shown in Fig. 5, but it is an important control. Furthermore, the authors point out that at 1 μM of pITIM-pITSM there is an increase in the activity of SHP-2, can the authors estimate the cytoplasmic concentration of SHP-2?

Altogether, while it is clear from the work here described by Patsoukis et al that the SHP-2 has the biophysical capacity to interact with two pTyr PD-1 ITSM from two different PD-1 molecules, two broad questions remain: how likely is this to happen? And second, how physiologically relevant is this observation? To better answer this, there are a couple of experiments that appear experimentally straightforward and which may contribute towards a better understanding of the PD-1/SHP-2 binding and activation mechanism in the context of this particular study: 1) what is the pITIM contribution (avidity) to the SHP-2 binding in the context of an existing pITSM/SH2 interaction? and 2) can the pITSM alone induce SHP-2 activation at physiologically relevant concentrations (even if low)?

Overall the methods section seems clear and the statistical analysis appropriate.

Reviewer #3 (Remarks to the Author):

In this manuscript, the authors present a comprehensive and compelling body of evidence that demonstrates that SHP-2 activation occurs through by dimerization of two PD-1 molecules. The findings are novel and potentially very important as new molecular interactions have been identified that could be targeted with therapies. The manuscript is long but very well written and insightful throughout. The figure layout, experimental rigor and statistical analysis are outstanding. The only concern is that the model apparently proposes PD-1 activation without interactions with known PD-1 ligands such as PD-L1. Thus, in the discussion, the authors need to consider how the model fits with PD-L1/PD-L2 ligation.

We would like to thank the reviewers for the careful consideration of our manuscript and their thoughtful suggestions for the improvement of our work. All the comments of the reviewers have been taken into consideration, additional work has been performed and the manuscript has been revised accordingly. All the changes and revisions are highlighted in the revised manuscript. Below is a point-by-point response to the reviewers' comments.

Reviewer #1 (Remarks to the Author):

Patsoukis et al Interaction of SHP-2 SH2 domains with PD-1 phospho-ITSM bridges two PD-1 molecules and can induce SHP-2 activation. This is an interesting study which provides new insights the PD-1 inhibitory receptor, which in important therapeutic target that has attracted huge interest in recent years. The authors use a range of cellular and biochemical approaches to support a model in which two phosphorylated PD-1 receptors simultaneously bind to and activate a sing SHP2 molecule. Their findings help reconcile previously puzzling results and raise interesting questions regarding whether and how PD-1 is dimerized upon ligand engagement. The study is well written with few if any errors, and the Figures are clear and convincing.

Some minor points:
1) Line 108/109 it states that the '..PD-1: SHP-2 interaction was induced by CD3 ligation with or without CD28 costimulation'. No experiments are shown where there is no anti-CD28 antibody included in the stimulation.

A: We included these results in Supplementary Figure S4.

2) Line 142-145: The sentence "Importantly, PD-1 ITSM-Y248F disrupted the interaction of PD-1 with either SHP-2 mutant (Fig. 2a, bottom panel and Supplementary Fig. S5a, bottom panel), suggesting that binding of each SH2 domain of SHP-2 with PD-1 when co-expressed in live cells depends on PD-1 ITSM-Y248" seems illogical. Since, as noted in the previous sentence, neither SHP-2 mutant bound WT PD-1, surely their lack of binding to PD-1 ITSM-Y248F tells you nothing extra.

A: We agree with the reviewer and we modified this description in the text (lines 144-147 in the revised manuscript).

3) Lines 153-7: "Consistently, compared with a peptide containing both phosphorylated tyrosines of the native PD-1 cytoplasmic tail (Fig. 2c, top panel), electrophoretic mobility shift of t-SHP-2 was preserved by a peptide containing phosphorylation of Y248 (PD-1cyto-ITIM-pITSM) but not by a peptide containing phosphorylation of Y223 (PD-cyto-pITIM-ITSM) (Fig. 2c middle and bottom panel)." In fact, looking at Fig. 2c, it seems that the pITIM, while not enough on its own for binding, does seem to enhance binding mediated by pITSM.

A: We agree with the reviewer and, for this reason, we studied this particular point further by assessing affinity of single vs. doubly phosphorylated peptide by ITC. These data are shown in Figure 3c-d and described in lines 218-221 of the revised manuscript.

4) Lines 192-3: "The affinity of each individual SH2 domain is negligible compared with the calculated affinity of SHP-2-full length (N-SH2 KD=2.2 μM, C-SH-2 KD=161 μM, SHP-2 KD=0.0197 μM) (Supplementary Fig. S7a, b, c)." These Kd values do not seem to match the raw data shown in Fig. 7b and c. These data clearly show that the C-SH2 single domain is binding to a comparable level than the N-SH2 single domain when injected at similar

concentrations, so it cannot be correct that the affinity of the C-SH2 single domain is 75 fold lower. I suspect they have obtained unreliable results because they have tried to fit ka and kd values and calculated the Kd. This is unreliable with low affinity interactions with fast kinetics. They should use a plot of steady state binding versus concentration to determine the Kd for these interactions.

A: We agree completely with the referee that individual Kd than Ka determinations may not be appropriate for the observed binding kinetics of SHP-2 N-SH2 and C-SH2 domains to ITSM-pY248 for the reasons the referee suggests. We have replotted the data from steady state conditions as Req against concentration then performed Steady State analysis using the fitting model within the BIAEval package to determine the K_A (and derived K_D) in addition to a Scatchard transformation to determine K_D (revised Supplementary Fig. S8c-f). Note that, as described by van der Merwe (Surface plasmon resonance. P. Anton van der Merwe. Protein-ligand interactions: hydrodynamics and calorimetry, Volume 1, pages 137-170, Oxford University Press: New York, NY, USA, 2001), Scatchard analysis reveals a non-linear "concave upwards" profile for binding of the N-SH2 domain. Such a profile may have a multiplicity of causes all beyond the scope of analysis in this article but none of which impact the basic conclusion that the binding of both individual N-SH2 and C-SH2 domains exhibit a >1.75 log reduced affinity on comparison with full-length SHP-2.

5) What is the relationship between Fig 5f and S12, which look very similar? The authors should clarify this and perhaps consider omitting S12.

A: The data shown in Fig. 5f and Fig. S12 (now Fig. S13 in the revised manuscript) are the same, as stated in the text. Fig. 5f is showing the changes in IL-2 production as % of the maximum IL-2 amount produced by each cell line, Fig. S13 shows the absolute IL-2 amounts produced in each experimental condition. Presenting the results as % of maximum IL-2 production (Fig. 5f) is more helpful to understand the effects of the different PD-1 than presenting only the absolute values (Fig. S13) because each cell line has a different maximum IL-2 production capacity.

Reviewer #2 (Remarks to the Author):

The work by Patsoukis et al is focused on the early mechanisms of PD-1 signaling via SHP-2 recruitment and activation. The topic is of course of interest to a wide audience, is timely and has the potential to impact translational work.

PD-1 contains two tyrosine-based structural motifs, and ITIM and an ITSM. Several studies have now shown convincingly that PD-1 phosphorylation (pTyr) recruits SHP-2, a cytoplasmic phosphatase, which then dephosphorylates multiple other transmembrane receptors. Some reports also have suggested a role for a PD-1/SHP-1 interaction, but I largely agree with the authors that SHP-2 appears to be the key player, based on the published data.

Upon PD-1 activation, a yet unexplored mechanism leads to pTyr of its two Tyr-based signaling motifs, ITIM and ITSM, which results in SHP-2 recruitment. The work by Patsoukis et al revolves around a fundamental second step: SHP-2 activation. Once SHP-2 is recruited to PD-1, via interaction of its SH2 domains with PD-1 pTyr, SHP-2 catalytic phosphatase domain is released from an auto-inhibitory conformation resulting in activation of the phosphatase activity. The mechanism of SHP-2 activation has been extensively studied. SHP-2 contains two tandem SH2 domains, N-term and C-term, followed by a C-terminal protein tyrosine phosphatase (PTP) domain. An intramolecular interaction between N-SH2 and the PTP leads to reduced PTP activity.

Binding of N-SH2 to a target pTyr has the potential to release the PTP from its intramolecular interaction and thereby enhance PTP activity. The authors very clearly explain all of this in the introduction.

The authors move on to investigate in more detail the interaction properties between PD-1 and SHP-2 and focus on two key aspects. First, the authors conclude that SHP-2 bridges two PD-1 molecules via the pTyr Y248 PD-1 residue (using a luminescence-based dimerization reporter system), in other words, SHP2 would dimerize PD-1 upon PD-1 pTyr. Secondly, the authors also provide more evidence for the SHP-2 interaction with PD-1 Y248 by using SPR and ITC. The measurements that support the second observation are robust but not novel, while the data that supports the dimerization of PD-1 upon SHP-2 recruitment is, I found, incomplete and inconclusive at this stage. The novelty of this work lies in this newly proposed mechanism of PD-1 SHP-2-induced dimerization, however the work feels slightly rushed and premature.

A: We appreciate the comment of the reviewer that the novelty of our work lies in the newly proposed mechanism of PD-1 SHP-2-induced dimerization and that our measurements that support the role of PD-1 Y248 in the interaction with SHP-2 are robust. However, we respectfully disagree that these studies are not novel. To our knowledge, our studies are the first to investigate the role of the individual SH2 domains of SHP-2 in the interaction with PD-1 Y248, by using mutagenesis of the active site of each SH2 domain. In addition, our studies are the first to investigate the stoichiometry of PD-1: SHP-2 interaction. Again, to our knowledge none of the previous relevant studies have investigated these issues.

Main concerns:

1)- In Figure 1 and 2 (and accompanying supplementary figures) the authors nicely show that the SHP-2/PD-1 interaction is largely mediated by PD-1 ITSM-Y248F, in good agreement with previous reports. The authors move on to investigate the contribution of the N-SH2 and C-SH2 for the PD-1/SHP2 interaction by performing pull-downs with PD-1 and R32A or R138A SHP-2 mutants. From these experiments the authors conclude that both domains are required for SHP-2 to interact with PD-1. The authors thus propose a model where both SH2 domains interact with pY248, whereby the N-SH2 domain binds one PD-1 molecule and the C-SH2 domain binds another PD-1 molecule also via the pY248. The authors conclude that this hypothesis explains why the Y248F mutation on PD-1 eliminates interaction with either SHP-2 WT or both SHP-2 SH2 mutants. Importantly, in data shown in Supplementary Fig. S7b, c, and which I think should be in the corresponding main Figure 3, the authors show that the affinity of the N-SH2 domain to pY248 is 75-fold higher than that of the C-SH2 domain. Notice that the affinity of the N-SH2 is (according to authors "negligible") 100-fold lower than the affinity of the SHP-2-full length. The experiment is however incomplete, a second set of measurements should have been made with doubly pTyr PD-1 peptide, pY223 and pY248. Is it possible that binding to pY223, once the stronger N-SH2 binding to pY248 is established, can further stabilize the PD-1/SHP-2 interaction? Notice that even the extremely weak C-SH2 interaction with pY248 (KD=161 µM) contributes with a 100-fold increase in affinity over N-term alone (N-SH2 KD=2.2 µM V WT SHP2 KD=0.0197 µM). It is possible that a weak pY223 can stabilize the binding to WT SHP-2, once one of the SH2 domains is bound to pY248. This is important because if that is the case then an intra-molecular interaction between the SH2s of SHP-2 and PD-1 is much more likely to occur than an interaction between the SH2s of SHP-2 and two PD-1 molecules diffusing independently and particularly when one of the interactions (that mediated by the C-term SH2) is so weak, > 150 µM. In other words, the authors clearly show that the interaction between SHP2 SH2 is mediated by Y248; Y223 interaction is negligible to non-existent on its own, but what about Y223 in the context of Y248? Intramolecular avidity

effects can drastically increase weak affinities, and as such this possibility should be accounted for. Given that this experiment was made for the ITC and that it showed that one PD-1 molecule can bind to SHP-2 using both pTyr 223 and 248, it would be important to include the same measurement in the context of the SPR work as well. Otherwise the authors are providing two conflicting pieces of data and it may appear that one result is being favored over the other.

A: In this comment, the reviewer is making various points: a) One of these is related to the affinity of the individual SH2 domains for binding to PD-1 Y248, and we agree with its importance. This issue is relevant to the comment #4 of reviewer 1. As mentioned in response to reviewer 1, we performed additional studies to clarify the binding properties and affinities of the individual SH2 domains to PD-1 Y248. These studies are described in the revised SPR section (lines 191-198 of the revised manuscript) and shown in the revised relevant supplementary figure, which is now Fig. S8. We kept these data in a supplementary figure because main Figure 3 is already busy with results and because we generated additional data that we included in supplementary Fig. S8. However, if the editor prefers to move these results from Supplementary Fig. S8 to main Fig. 3, we can do it.

b) A second point that the reviewer is making in this comment is that Y223, although incapable on its own of sustaining interaction with SHP-2, might contribute to the SHP-2 interaction with PD-1 Y248. We totally agree with this and we have performed additional studies to investigate this issue by using doubly phosphorylated PD-1 phosphopeptide. For this purpose, we have used ITC, which provides the ability to assess simultaneously stoichiometry and affinity of interaction. We respectfully disagree that SPR and ITC provide two conflicting pieces of data. Each of these assays is employed purposely to serve a different goal in our studies and to generate complementary results. We used SPR to study the binding properties of the individual PD-1 tyrosines to SHP-2 full length and to the individual SH2 domains. Using doubly phosphorylated PD-1 peptides in SPR would not allow us to dissect whether the interaction occurs through binding of SHP-2 to both PD-1 phosphotyrosines or to the same phosphoryrosine (i.e. Y248) on two different molecules. To address this, we chose to employ ITC, which allowed us to assess stoichiometry and affinity of interaction simultaneously. These studies are described in detail in lines 199-224 of the revised manuscript.

2)- In the intracellular, cytoplasmic, environment KCl concentration is higher than NaCl. Given the low affinities the authors are reporting here (> µM), would it be possible to consider repeating some of the measurements in a buffer similar to: 10mM Hepes, pH 7.4, 146 mM KCl, and 4 mM NaCl 1mM DTT ? John Kuriyan and others have shown that for largely electrostatic mediated interactions, such as those established between SH2/pY, the salt composition of the buffer can have an important effect.

A: We agree that the buffers used in biochemical assays might not represent precisely the intracellular cytoplasmic environment. In our studies we used standard in vitro methods to compare the tested interactions under commonly used buffer conditions. Although the salt composition of the buffer might have an effect on the in vitro interaction, our SPR studies showed good SHP-2 FL/pITSM interaction that can serve as a positive control, and for this reason, interpreting degrees of binding in the same buffer allows appropriate comparisons. Further, although changing the salt composition of the buffer might have an effect on the in vitro interaction, such change would not recapitulate the conditions of the intracellular environment. For these reasons, we believe that testing interactions in various buffer conditions does not have direct biological relevance and is beyond the scope of our present work.

3)- The NanoBiT proximity assay has a very important caveat which the authors do not seem to

consider. How confident are the authors that the luminescence signal is from the cell membrane and not from, for example, the ER? A FRET reporter, whereby the source of the signal could be confirmed could be useful. Regardless of the reporter used it is important to confirm the main source of the signal, particularly because the proteins under study are being overexpressed. This is even more important when some of the data appears to be slightly puzzling. For example, dimeric recombinant PD-L1 binding alone does not seem to induce a significant increase in PD-1 dimerization. Expression of kinase active Fyn seems to be required for dimerization. But shouldn't PD-L1 dimer be enough? This raises questions in terms of how much signaling is derived from surface membrane PD-1 and/or what is the assay sensitivity? It is also concerning that the expression of the PD-1Lg and PD1SM nanobit appears very low (Fig. S9a), which may hint at problems in protein stability. WT PD-1 expresses to very high levels (see Fig S2 and S4), while the nanobit PD1 constructs show extremely low levels of expression, even in HEK-293s. Perhaps the authors could perform an intracellular staining of PD-1 in HEK and Primary cells to confirm the ratio of surface:intracellular PD-1-nanobit construct.

A: a) The key point of the reviewer in this comment is to identify the source of the signal and confirm that PD-1 dimerization occurs at the plasma membrane and not in another site of the cell, for example the ER. We performed new studies with confocal microscopy, which provided evidence that PD-1 dimerization occurs at the plasma membrane. These new results are shown in Figure 4f-g and described in the relevant result section, lines 279-286 of the revised manuscript.

b) A second point that the reviewer mention in this comment is related to the role of PD-1 phosphorylation in the generation of PD-1 ligand-mediated dimer. We have provided interpretation and discussion of these findings in lines 475-489 of the revised manuscript.

c) A third point in this comment is related to the expression level of PD-1 in the NanoBit experiments. Expression of transfected PD-1 NanoBiTs was kept purposely low in these studies to avoid a high background signal that might be generated from the high density of closely localized PD-1 molecules on the cell surface. Such conditions would not allow assessment of experimentally-induced dimerization. We have performed flow cytometry assays to examine expression of PD-1 in transfected HEK-293 cells and primary T cells, and side-by-side comparison with PD-1 expression in primary human T cells before and after stimulation. These results are shown in Supplementary Fig. S10. The cells in Fig. S2 that the reviewer stated to show high levels of WT PD-1 expression are stably transfected Jurkat cells and serve different purposes. These cells were not used for any of the dimerization assays.

4)- Later on, the authors claim that SHP-2 activation requires binding of both SH2 domains to "release" the inhibitory state of the catalytic PTP site. This may not be entirely correct. Hayashi et al, Cell Reports, 2017, show that a single, monovalent, interaction between a pY peptide and the N-term SH2 in SHP-2 is necessary and sufficient to induce strong enzymatic activation of SHP-2 using a particular peptide (https://www.sciencedirect.com/science/article/pii/S2211124717312226?via=ihub). Have the authors tested the PTP activity in the presence of pITSM alone? This is not clear from the data shown in Fig. 5, but it is an important control. Furthermore, the authors point out that at 1 μM of pITIM-pITSM there is an increase in the activity of SHP-2, can the authors estimate the cytoplasmic concentration of SHP-2?

A: It has been known for many years that binding of monophosphorylated peptides to SHP-2 N-SH2 can induce some degree of activity at high peptide concentrations. In fact, before it was discovered how PTP activity of SHP-2 is regulated, it was thought that only a single

phosphotyrosine binding was required for activation of SHP-2 PTP activity. We have cited several of these publications in our manuscript (e.g. ref #18, 19, 42, 43). Also, as the reviewer requested, we used single PD-1 phosphopeptides to assess their capacity of inducing SHP-2 activation. These results are shown in Figure 5a.

The second point of the reviewer is what the cytoplasmic concentration of SHP-2 is. The reviewer links this question to our in vitro finding that pITIM-pITSM can induce low levels of SHP-2 activation at concentrations above 1 µM. We believe that estimating the total cytoplasmic concentration of SHP-2 is not helpful to understand the biological significance of the in vitro measured ability of the PD-1 peptides to induce SHP-2 activation for several reasons: First, the concentration of 1 μM in our studies refers to the PD-1 phosphopeptide -not to SHP2. Second, the concentration of interacting proteins at the subcellular compartment in which such interactions occur and at the time they occur, is dramatically different from the total steady state cytoplasmic concentration of such proteins. For instance, SHP-2 will become highly concentrated in a specific subcellular compartment in which SHP-2: PD-1 binding occurs, such as the plasma membrane, and estimating cytoplasmic concentration of SHP-2 will not provide any information about its concentration in the compartment in which such interaction occurs. Third, the concentration assessed in our studies involves a peptide -not a full-length protein, as those present in the cells. Fourth, the in vitro PTP assay includes only the two interacting components of interest (SHP-2 and PD-1 phosphopeptide), whereas in the cell, each of the corresponding full-length proteins has multiple partners. Thus, estimates of cytoplasmic concentration of SHP-2 would not provide any meaningful information regarding the SHP-2: PD-1 interaction.

5) Altogether, while it is clear from the work here described by Patsoukis et al that the SHP-2 has the biophysical capacity to interact with two pTyr PD-1 ITSM from two different PD-1 molecules, two broad questions remain: how likely is this to happen? And second, how physiologically relevant is this observation? To better answer this, there are a couple of experiments that appear experimentally straightforward and which may contribute towards a better understanding of the PD-1/SHP-2 binding and activation mechanism in the context of this particular study: 1) what is the pITIM contribution (avidity) to the SHP-2 binding in the context of an existing pITSM/SH2 interaction? and 2) can the pITSM alone induce SHP-2 activation at physiologically relevant concentrations (even if low)?

A: These two concluding points of the reviewer have been addressed in our revised studies. The answer of the first point is shown in Figure 3c and d and the answer of the second point is shown in Figure 5a.

Overall the methods section seems clear and the statistical analysis appropriate.

Reviewer #3 (Remarks to the Author):

In this manuscript, the authors present a comprehensive and compelling body of evidence that demonstrates that SHP-2 activation occurs through by dimerization of two PD-1 molecules. The findings are novel and potentially very important as new molecular interactions have been identified that could be targeted with therapies. The manuscript is long but very well written and insightful throughout. The figure layout, experimental rigor and statistical analysis are outstanding. The only concern is that the model apparently proposes PD-1 activation without interactions with known PD-1 ligands such as PD-L1. Thus, in the discussion, the authors need

to consider how the model fits with PD-L1/PD-L2 ligation.

A: We thank this reviewer for the favorable comments about the significance of our findings and the very well written and insightful manuscript. We have addressed the very accurate point that reviewer raised regarding the involvement of PD-1 ligands in the process of PD-1 engagement and dimer formation. This has been included in the revised Figure 5g and in the discussion section of the revised manuscript, lines 475-489 and the relevant figure legend, lines 1164-1170.

REVIEWERS' COMMENTS:

Reviewer #1 (Remarks to the Author):

The authors have addressed the points that I raised.

Reviewer #2 (Remarks to the Author):

I would like to thank the authors for the thoughtful point-by-point rebuttal to my previous questions. I find the manuscript to be significantly improved with the newly added experiments and the modifications to the main text. The main findings seem now to be robustly supported by experimental observations and as such I have no further reservations.
